# A Trifunctional Linker for Palmitoylation and Peptide and Protein Localization in Biological Membranes

**DOI:** 10.1002/cbic.201900655

**Published:** 2020-01-10

**Authors:** Łukasz Syga, Reinder H. de Vries, Hugo van Oosterhout, Rianne Bartelds, Arnold J. Boersma, Gerard Roelfes, Bert Poolman

**Affiliations:** ^1^ Department of Biomolecular Chemistry and Catalysis Stratingh Institute for Chemistry University of Groningen Nijenborgh 4 9747 AG Groningen The Netherlands; ^2^ Department of Biochemistry Groningen Biomolecular Sciences and Biotechnology Institute and Zernike Institute for Advanced Materials University of Groningen Nijenborgh 4 9747 AG Groningen The Netherlands; ^3^ DWI Leibniz Institute for Interactive Materials Forckenbeckstrasse 50 52074 Aachen Germany

**Keywords:** biological membranes, lipid phase separation, membrane partitioning, palmitoylation, trifunctional linker

## Abstract

Attachment of lipophilic groups is an important post‐translational modification of proteins, which involves the coupling of one or more anchors such as fatty acids, isoprenoids, phospholipids, or glycosylphosphatidyl inositols. To study its impact on the membrane partitioning of hydrophobic peptides or proteins, we designed a tyrosine‐based trifunctional linker. The linker allows the facile incorporation of two different functionalities at a cysteine residue in a single step. We determined the effect of the lipid modification on the membrane partitioning of the synthetic α‐helical model peptide WALP with or without here and in all cases below; palmitoyl groups in giant unilamellar vesicles that contain a liquid‐ordered (L_o_) and liquid‐disordered (L_d_) phase. Introduction of two palmitoyl groups did not alter the localization of the membrane peptides, nor did the membrane thickness or lipid composition. In all cases, the peptide was retained in the L_d_ phase. These data demonstrate that the L_o_ domain in model membranes is highly unfavorable for a single membrane‐spanning peptide.

## Graphical abstract

**Attachment of lipophilic groups** is an important post‐translational modification of proteins. To study its impact on the membrane partitioning of hydrophobic peptides/proteins, we designed a tyrosine‐based trifunctional linker that allows facile incorporation of two different functionalities at a cysteine residue in a single step. Shown is a liquid‐disordered phase of a giant unilamellar vesicle (green semicircle), trifunctional linker (blue) connected to a fluorophore (black), DPPE lipid (brown), and protein.

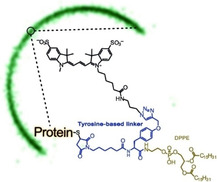

## Introduction

The traffic of proteins to the proper localization in the cell is necessary for their function. In many instances signal sequences determine the destination of a protein, be it the insertion within a membrane or translocation into the lumen of a compartment of a cell.^1^, ^2^, ^3^ Remarkably, changes of a single amino acid residue can change the localization of lipoproteins from the inner to the outer membrane of *Escherichia coli* and vice versa.^4^ Furthermore, reaching the correct compartment or target membrane is not necessarily enough for proper functioning of the protein. Biological membranes are heterogeneous in structure and localization within a specific membrane domain has been shown to affect the function of for example, Lymphocyte function‐associated antigen 1 and Toll‐like receptor 2, which are moved to more ordered domains upon binding of a substrate.^5^, ^6^, ^7^, ^8^, ^9^ The signal output of K‐Ras changes when a lysine residue is changed into glutamine.^10^ This modification altered the interaction of K‐Ras with anionic lipids and consequently the sorting of those lipids into nanodomains.^10^

Protein palmitoylation is a reversible post‐translational modification whereby one or more palmitic acid group(s) are attached to a cysteine residue (or more seldom, a serine or threonine residue), and this modification has been implicated in the localization of proteins within a given membrane.^11^, ^12^, ^13^, ^14^ The palmitic acid group changes the hydrophobicity of the complex and may drive its partitioning into a specific membrane domain.^14^ For example, the linker for activation of T cells (LAT) is enriched in the raft phase of cell‐derived vesicles when palmitoylated.^15^ In cells, the doubly palmitoylated H‐Ras protein is localized in a different compartment than the unpalmitoylated K‐RAS.^16^ In yeast, several amino acid permeases (AAPs) have a C‐terminal, amphipathic α‐helix that associates with the inner leaflet of the plasma membrane. In a subset of these proteins, for example the amino acid transporters Gap1 and Tat2, this C‐terminal helix is palmitoylated by a palmitoyl‐acyl transferase.^17^ The deletion of the amphipathic α‐helix does not affect the apparent localization of Gap1 and Tat2 but leads to diminished growth on non‐fermentable carbon sources.^18^ Overall, palmitoylation of membrane proteins is relatively widespread in biology, but the functional significance of this modification is in most cases far from clear.

Hydrophobic mismatch is also known as sorting principle for membrane proteins. When the hydrophobic part of a membrane protein or peptide and the lipid membrane have different thickness, the lipids surrounding the protein are distorted which comes with an energetic penalty.^19^, ^20^ Proteins preferentially reside in lipid domains with matching thickness, which leads to segregation of proteins with different hydrophobic thickness.^21^, ^22^, ^23^, ^24^

WALP peptides are classical transmembrane peptide models commonly used to study membrane protein/peptide behavior in vitro^25^, ^26^, ^27^, ^28^, ^29^, ^30^ and in silico.^31^, ^32^, ^33^ Their sequences consist of alanine and leucine repeats flanked by tryptophan residues. Series of WALP peptides with different length and sequences have been developed.^34^ Here, we use WALP derivatives to investigate the effect of palmitoylation on the localization of a hydrophobic model peptide within the lipid domains of synthetic membranes.

To study the effect of protein palmitoylation and hydrophobic matching, we designed a trifunctional linker to couple a peptide or protein to both a fluorophore and (a) palmitoyl chain(s). Trifunctional linkers and scaffolds are ubiquitous in chemistry and serve a great variety of purposes. Even the simplest linkers bearing three identical reactive groups can be used, taking advantage of stochastic coupling or clever (sub‐stoichiometric) introduction of the test molecules.^35^, ^36^, ^37^ Trifunctional linkers bearing three different reactive groups are more challenging to synthesize.^38^, ^39^, ^40^, ^41^, ^42^ A very elegant option is a molecule based on a tri‐orthogonal “click” scaffold, combining inverse electron demand Diels–Alder (iEDDA) between a cyclooctyne and a tetrazine moiety with a copper‐catalyzed alkyne–azide click (CuAAC) reaction as well as maleimide coupling.^43^ The combination of two CuAAC click reactions with an aldehyde and/or activated ester coupling is an alternative option.^44^ Herein we report the synthesis of a tyrosine‐based trifunctional linker in which the palmitoyl chains, fluorophore and peptide are conjugated onto one scaffold via amide, CuAAC and maleimide coupling, respectively. As membrane model system to test the partitioning of hydrophobic peptides we used phase‐separating giant unilamellar vesicles (GUVs). The chosen lipid compositions separate the GUV membrane into liquid‐ordered (L_o_) and liquid‐disordered (L_d_) phases.^45^, ^46^ In addition, we studied the effect of palmitoylation and hydrophobic mismatch in this system. We find that the lipid modification of the hydrophobic peptide does not affect its membrane partitioning and that the L_o_ phase is disfavored for all molecules and lipid compositions tested.

## Results

### Synthesis of tyrosine‐based trifunctional linker and characterization of palmitoylated and fluorophore‐coupled peptides

**Scheme 1:**
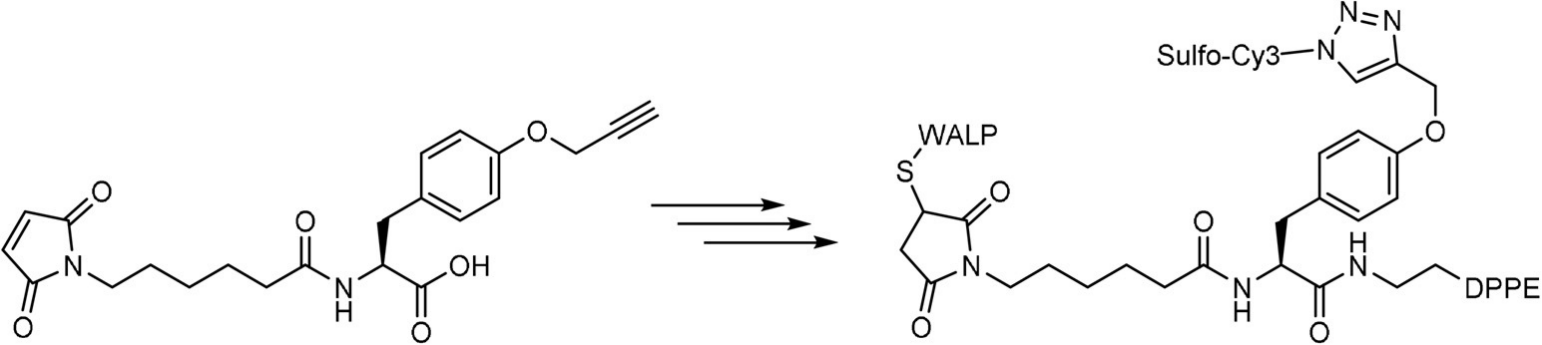
Trifunctional linker used to conjugate 1) DPPE via amide coupling, 2) Sulfo‐Cy3 azide via CuAAC, and 3) WALP via maleimide coupling.

Amino acids are a good starting point for the development of a trifunctional linker, as most naturally occurring amino acids already contain three functional groups. For the trifunctional linker designed here, a maleimide was introduced on *O*‐propargyltyrosine, followed by an amide coupling with phospholipid 1,2‐dipalmitoyl‐*sn*‐glycero‐3‐phosphoethanolamine (DPPE) to mimic two palmitoyl moieties. The propargyl allows CuAAC with a fluorescent dye (Sulfo‐Cy3 azide), while the maleimide undergoes a Michael reaction with an introduced cysteine in the WALP23 peptide (sequence: GCGWW(LA)_8_LWWA; Scheme 1).

**Scheme 2:**
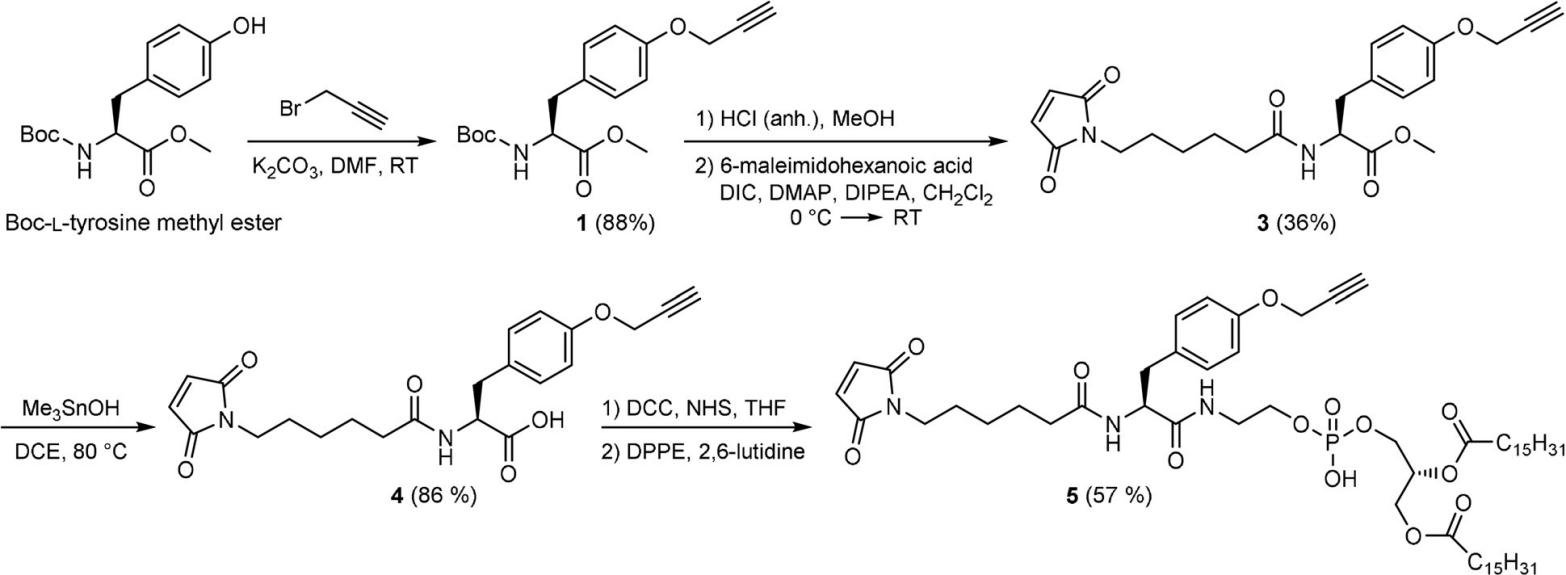
Synthetic route toward scaffold **5**.

We developed a synthetic route starting from Boc‐tyrosine methyl ester (Scheme 2). First, a propargyl moiety was installed on the phenolic‐OH after which **1** was obtained in high yields (Supporting Information). Boc‐deprotection followed by amide coupling with 6‐maleimidohexanoic acid, using DIC as a coupling reagent, resulted in amide **3** in moderate yields. Using the Nicolaou ester hydrolysis reaction,^47^ employing trimethyl tin hydroxide, the free acid (**4**) was obtained in high yields. In the next step, DPPE was introduced on the carboxylic acid in a two‐step procedure. First, an activated ester of **4** was generated in situ, followed by rapid removal of the insoluble urea side products. The activated ester was added to the solution containing DPPE, resulting in the phospholipid‐modified scaffold **5** in good yield.

**Scheme 3:**
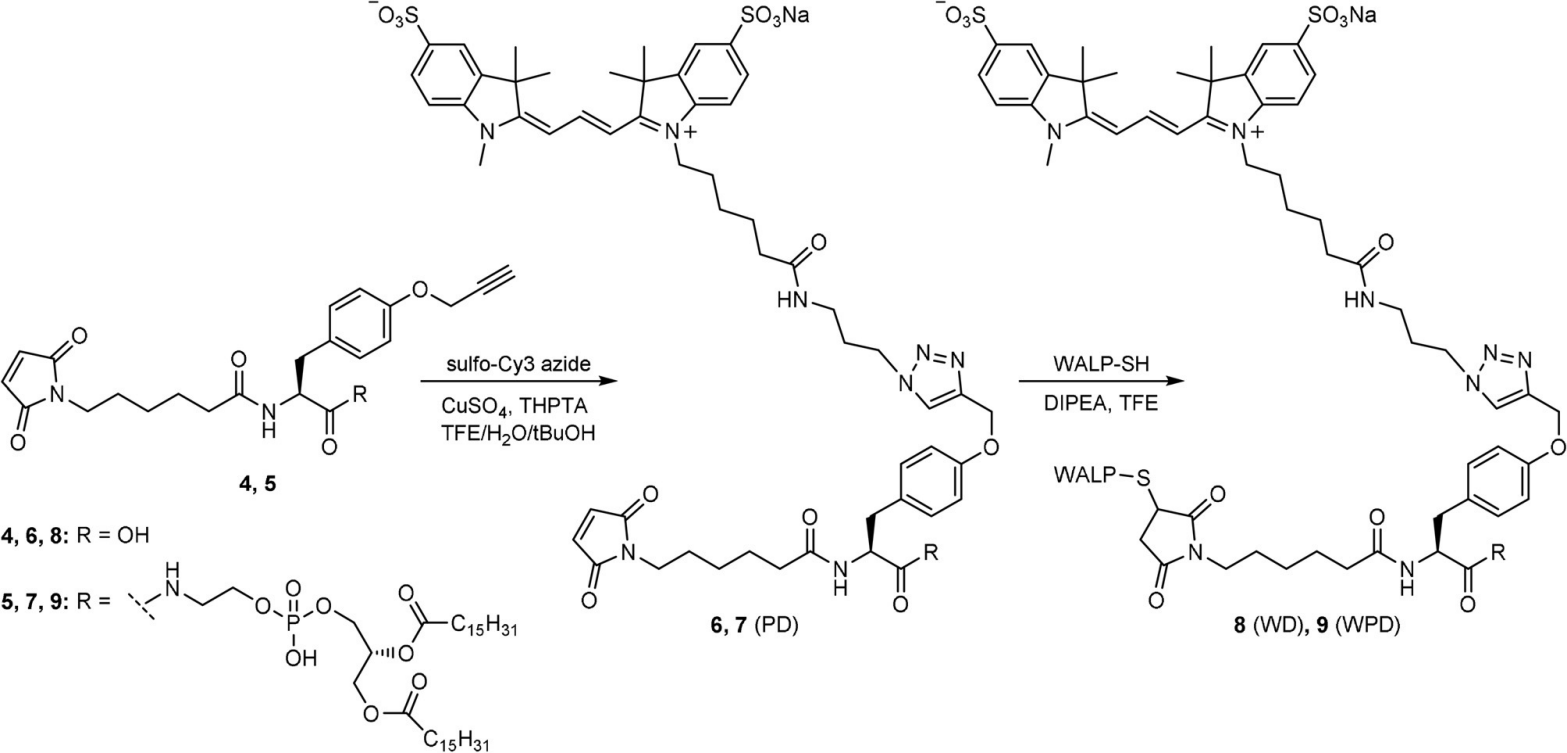
Sequential conjugation of Sulfo‐Cy3 and WALP on scaffolds **4** and **5**.

The functionalization of the DPPE‐modified linker **5** with Sulfo‐Cy3 azide via CuAAC gave the palmitoyl‐dye (PD) product **7** in 24 % yield after preparative HPLC purification (Scheme 3). For control experiments, free acid **4** (without DPPE) was also labeled with Sulfo‐Cy3 using the same protocol, after which control conjugate **6** was obtained in 47 % yield. Subsequent conjugation of **6** and **7** with WALP followed by preparative HPLC purification afforded the WALP‐dye (WD, **8**) and WALP‐palmitoyl dye (WPD, **9**) constructs, respectively (Scheme 3).

### Partitioning of the tyrosine‐based trifunctional linkers and WALP in phase‐separating GUVs

**Figure 1:**
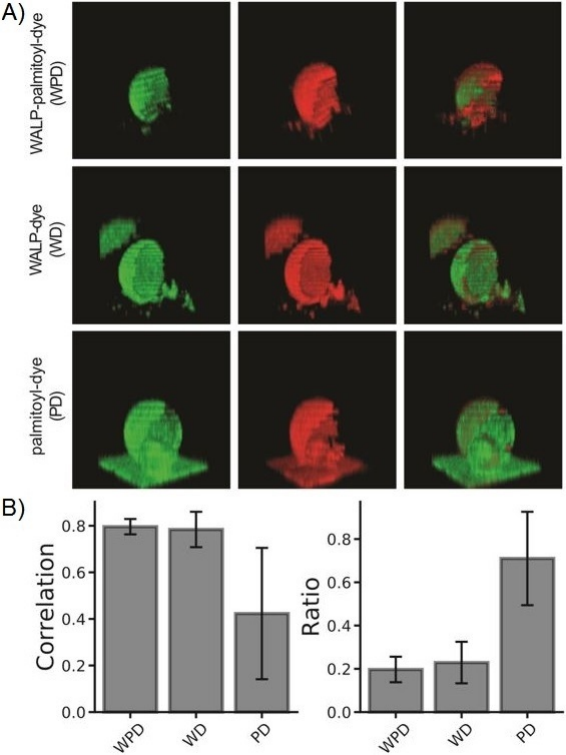
Partitioning of the constructs within the membrane domains of phase‐separating GUVs. A) 3D reconstructions of GUVs with the SulfoCy3‐labeled constructs in green and the L_d_ marker in red (Atto655). B) Pearson correlation for the construct relative to the marker (left), and the ratio of the molecules for L_o_ and L_d_ (L_o_/L_d_ ratio; right). Error bars represent the standard deviation of three separate preparations of the GUVs.

We determined the localization and partitioning of the WALP peptide with and without palmitoyl moiety in phase‐separating GUVs composed of DPPC, DOPC, and cholesterol in 4:3:3 molar ratio (Figure 1 A). This lipid composition results in membranes with distinct phases, with most of the DPPC and cholesterol in the so‐called L_o_ phase and most of the DOPC in the L_d_ phase.^48^ The constructs WALP‐palmitoyl‐dye (WPD; construct **9**), WALP‐dye (WD; construct **8**), and palmitoyl‐dye (PD; construct **7**) were used. Each construct was imaged with Atto655‐DOPE as marker of the L_d_ phase. The Atto 655 dye displays minimal interaction with the membrane.^49^ The Pearson correlation coefficient for the constructs (**7**, **8** or **9**) relative to the L_d_ marker was calculated to signify the preference of the modified peptide in either of the phases. A Pearson's correlation coefficient of 1 indicates that the two signals increase and decrease identically, whereas a value of 0 indicates a random relation between the signals. A negative value would indicate anticorrelation of two signals. We also determined the ratio of the molecules for the two phases (L_o_/L_d_), using the mean fluorescence in each phase as a measure of the partition coefficient of the constructs. The palmitoyl lipid (PD) has a slight preference for L_d_ (L_o_/L_d_ ratio of 0.71±0.21), whereas the constructs with WALP (WPD and WD) partition almost exclusively in L_d_ (Figure 1 B). To eliminate the possibility that the localization of WPD and WD in L_d_ is biased by aggregation of the peptide, experiments at 100‐fold lower concentration of the functionalized peptides were performed. The results were the same, and clearly, the palmitoyl moiety is not sufficient to transfer the WALP peptide from the L_d_ to the L_o_ phase (Figure S1 in the Supporting Information).

### Lipid acyl chain saturation, not acyl chain length, determines localization of WALP

**Figure 2:**
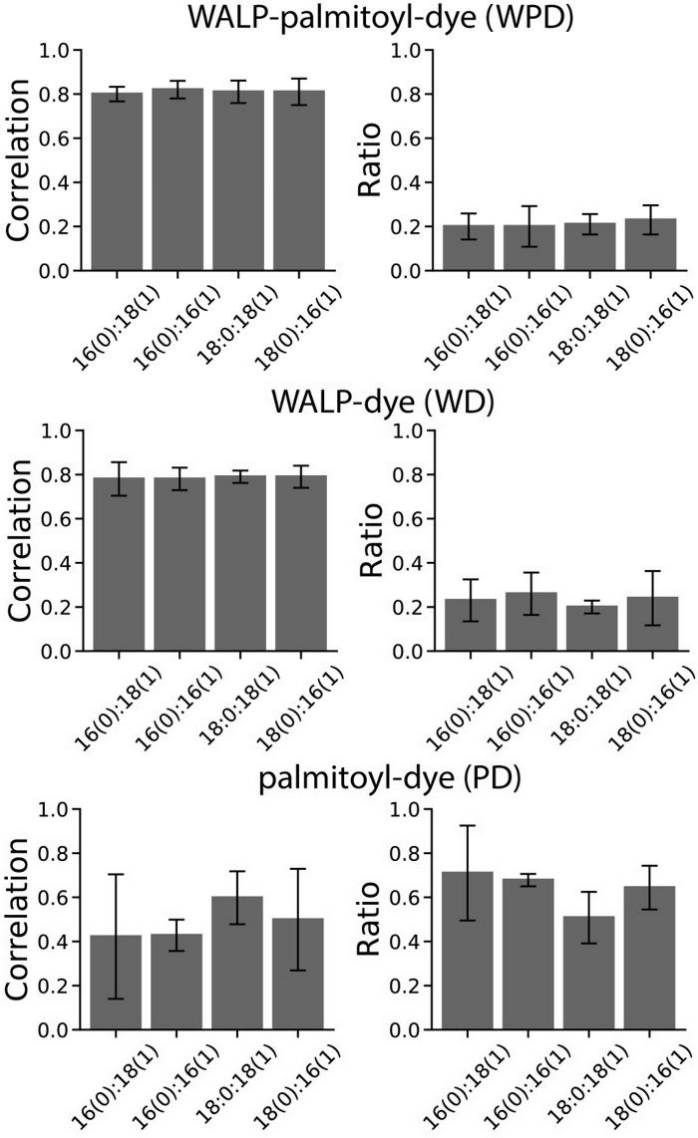
Effect of lipid composition of the GUVs on the localization of WALP constructs with or without a palmitoyl group. The Pearson correlation for the construct relative to the marker (left), and the ratio of the molecules for L_o_ and L_d_ (L_o_/L_d_ ratio; right) are shown. Error bars represent the standard deviation of three separate preparations of the GUVs.

Next, we investigated the effect of membrane thickness on the partitioning of the constructs. To this end, we varied the length of the acyl chains of the lipids, yet maintaining distinct L_d_ and L_o_ phases. We prepared GUVs composed of cholesterol plus DPPC (16:0)/DOPC (18:1), and GUVs made from cholesterol plus PC with different acyl chain composition: 16:0/16:1, 18:0/18:1, and 18:0/16:1. For these mixtures we assume that the majority of DPPC (16:0) and DSPC (18:0) is in L_o_ and the majority of DOPC and 16:1 PC in L_d_.^50^ Additionally, mixtures with 14:0, 14:1, and 22:0 PC lipids were tested but conditions resulting in phase‐separation were not found. All the constructs co‐localize with the L_d_ marker used, which in all cases indicates a preference for the unsaturated lipid, independent of its acyl chain length (Figure 2).

The localization of a molecule in the L_d_ or L_o_ phase of a membrane affects its lateral diffusion. Because the mobility of molecules in the L_d_ phase is much faster than in the L_o_ phase_,_ 45 we determined the lateral diffusion coefficient of Atto655‐DOPE in GUVs prepared from different lipid compositions. The diffusion coefficients (D) were: 1.53±0.41 (*n*=12), 3.11±0.55 (*n*=8), 1.10±0.256 (*n*=10), and 1.04±0.154 (*n*=5) μm^2^ s^−1^ (±SD) for the 16:0/18:1, 16:0/16:1, 18:0/18:1, and 18:0/16:1 mixtures, respectively. These values are consistent with lipid diffusion in the L_d_ phase and at least an order of magnitude faster than what is expected for L_o_.^45^, ^51^ Thus, we conclude that in all these lipid mixtures the Atto655‐DOPE localizes in the L_d_ phase of GUVs.

### The length of WALP does not affect the membrane localization

**Figure 3:**
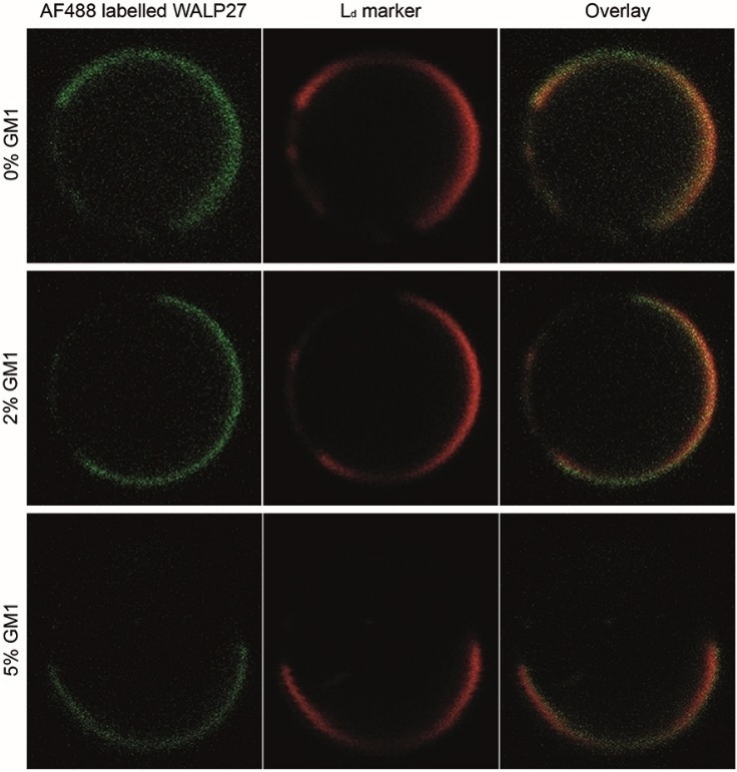
Confocal images of giant‐unilamellar vesicles (GUVs). Hydrophobic mismatch and GM1 do not alter the localization of WALP peptides as WALP27 (like WALP23) partitions preferentially in the L_d_ phase. WALP27 was labeled directly with AF‐488 (Supporting Information); DiD was used as L_d_ marker.

Next, we increased the length of the WALP peptide to better fit the hydrophobic thickness of the L_o_ phase, which is 0.7 to 1 nm larger than in the L_d_ phase.^52^, ^53^, ^54^ WALP27 is four amino acids longer than the WALP‐23, which results in an increase in the length of the hydrophobic part of 0.6 nm.^55^ We also determined the partitioning of WALP27 in the presence of up to 5 mol % of GM1, which induces tighter lipid packing and is thought to be a major component of rafts in mammalian cells.^56^ GM1 is relatively abundant in plasma membranes of the central nervous system of mammals.^57^ WALP27 labelled with AlexaFluor 488 (Supporting Information) co‐localized with the L_d_ marker DiD in the phase‐separating GUVs, irrespective of the presence of GM1 (Figure 3).

## Discussion

We report the synthesis and use of a trifunctional linker to study the localization of membrane proteins and peptides in phase‐separating giant unilamellar vesicles (GUVs). The linker allows great flexibility in connecting a triage of molecules such as fluorophores, peptides (or proteins) and other functionalities, such as lipid moieties. It is possible to selectively modify peptides (or proteins) with two different functional groups in a single step. Our synthetic approach of coupling two palmitoyl groups of DPPE to a membrane peptide via the trifunctional linker differs from biological systems where the lipid moieties are attached to cysteine on the peptide.

WALP and derivative peptides are commonly used as α‐helical models of membrane proteins and their interaction with lipid membranes has been studied extensively.^58^, ^59^, ^60^ The membrane localization and structure of WALP have been studied in silico and in vitro, using phase‐separating GUVs. With both approaches, WALP localizes in the L_d_ phase of the membranes.^26^, ^28^, ^61^, ^62^ Consistent with these findings, WALP is found in the detergent‐soluble fraction of phase‐separating large unilamellar vesicles,^63^ which is analogous to the L_d_ phase of GUVs observed by optical microscopy.^64^, ^65^, ^66^, ^67^ We show that the partitioning of the WALP with trifunctional linker is identical to that of genuine membrane peptides. Additionally, WALP partitions in the L_d_ phase even when two L_o_‐favoring palmitoyl groups are added via the trifunctional linker. The construct without WALP (PD) is distributed almost equally between L_o_ and L_d_ domains. This result was expected as even GM1, one of the defining components of rafts,^5^ needs to be complexed with cholera toxin to stain the L_o_ phase specifically.^68^ Adding two palmitoyl tails to the WALP peptide lowers the energy barrier for entry into L_o_ phase, but not enough for WALP to localize into the more ordered parts of GUVs with our tertiary lipid compositions. Hydrophobic mismatch created by changing the lipid composition of the membrane or the hydrophobic length of the WALP peptide does not alter the localization or partitioning significantly, irrespective of the presence of palmitoyl groups.

Palmitoylation has been shown to affect the membrane localization of LAT and hemagglutinin.^69^ LAT is localized in the L_o_ phase of giant plasma membrane vesicles, and the partitioning in L_o_ is diminished after depalmitoylation.^15^ However, LAT does not localize in the L_o_ of GUVs composed of DSPC (18:0; 33.3 mol %), DOPC (18:1; 31.7 mol %), DOPG (18:1; 1.6 mol %) plus cholesterol (33.3 mol %). In addition, palmitoylation of LAT does not affect the partitioning of the molecule in these vesicles,^70^ which is consistent with our findings on the localization of WALP with or without palmitoyl groups.

What could be the reason for the apparent inconsistency in the membrane domain partitioning of LAT and other membrane proteins or peptides? The liquid‐ordered and liquid‐disordered domains of membranes are qualitative descriptions of lipid ordering, and, depending on the actual lipid composition, a domain can be more, or less, ordered or disordered. We have attempted to address this point by varying the lipid composition of the membrane, while conserving microscopically observable L_o_ and L_d_ phases. We have lowered the energy barrier for entering the L_o_ domain by creating hydrophobic mismatch, but we always find WALP associated with L_d_. We cannot rule out that the transfer from L_d_ to L_o_ is more favorable in giant plasma membrane‐derived vesicles, consisting of hundreds of different lipid components,^69^ that phase separate with a smaller difference in lipid order between L_o_ and L_d_.^46^ We also note that the angle at which WALP crosses the membrane does not vary much with the membrane thickness.^27^, ^71^ In fact, in thin membranes with very high hydrophobic mismatch the peptides are no longer incorporated in the membrane rather than highly tilted.^71^ Finally, in vivo, factors such as accessible surface area can also affect the partitioning of membrane peptides and proteins;^72^ we have not investigated this aspect.

GM1, a ganglioside, is often used as raft marker^73^, ^74^, ^75^ and associated with the L_o_ phase.^76^, ^77^, ^78^ GM1 has been shown to interact with WALP and LAT, thereby favoring the partitioning of the peptides in the L_o_ phase, at least in coarse grained molecular dynamics simulations.^61^ All‐atom simulations contradict these findings and show depletion of GM1 near WALP.^79^ We note that the latter experiments were carried out in uniform phospholipid bilayers rather than phase‐separating membranes. GM1 could form small nanodomains inside the L_o_ phase,^77^, ^80^, ^81^ thereby preventing interaction with WALP. Clusters of GM1 have been found in cell membranes,^80^ but also in supported bilayers.^76^, ^77^, ^81^ In any case, direct interaction of GM1 and WALP in phase‐separating membranes seems unlikely, at least in our experimental system, because they are spaciously separated with GM1 in the L_o_ phase and WALP in the L_d_ phase.^62^, ^63^

## Conclusion

We present the design and synthesis of a new tyrosine‐based trifunctional linker, which enables conjugation of precious protein with two functional molecules in one step. To show the potential of our modular platform we study localization and partitioning of membrane‐embedded peptides. Contrary to our initial hypothesis, we find in GUVs, prepared from a variety of lipid mixtures, that a double palmitoyl moiety is not sufficient to change the partitioning of a single‐membrane spanner like WALP.

## Experimental Section

**Materials**: Lipids were obtained from Avanti Polar Lipids, Inc: 1,2‐dioleoyl‐*sn*‐glycero‐3‐phosphocholine (DOPC; product number 850375), 1,2‐dipalmitoleoyl‐*sn*‐glycero‐3‐phosphocholine (product number 850358), 1,2‐dipalmitoyl‐*sn*‐glycero‐3‐phosphocholine (DPPC; product number 850355), 1,2‐distearoyl‐*sn*‐glycero‐3‐phosphocholine (DSPC, product number 850365).

For immobilization of GUVs, we used glutaraldehyde from Sigma–Aldrich, product number 340855. APTES ((3‐Aminopropyl) triethoxysilane) was obtained from Sigma–Aldrich, product number 440140. WALP (GCGWW(LA)_8_LWWA) was purchased from Bachem. Sulfo‐Cyanine3 azide (Sulfo‐Cy3) was purchased from Lumiprobe. All other chemicals were reagent grade and obtained from various commercial sources. High precision coverslips (type #1.5H) were obtained from Ibidi GmbH (product no. 10812). Reactions were monitored by TLC Silica 60 (Merck Millipore), examined under UV (365 nm and 254 nm), and stained by KMnO_4_, ninhydrin, vanillin or H_2_SO_4_ in MeOH (1 %). Flash chromatography was performed on Silica gel 60 (0.040–0.063 mm) from Merck Millipore. ^1^H NMR spectra were recorded at 300 or 400 MHz and ^13^C NMR spectra were recorded at 75 MHz. The chemical shifts are reported in ppm relative to the residual solvent peak (CDCl_3_ at *δ*_H_=7.26 ppm, *δ*_C_=77.16 ppm). Yields of the dye constructs were based on UV absorption at 548 nm and the molar absorptivity coefficient of Sulfo‐Cy3 azide (*ϵ*=162 000 m ^−1^ cm^−1^ at 548 nm), not compensating for the presence of lipid or linker (assumed to not absorb in that region). HPLC was performed on a Shimadzu HPLC system equipped with LC‐20AD solvent chromatographs, a DGU‐20A3 degasser unit, a SPD‐M20A PDA detector, a SIL‐20A autosampler, a CTO‐20A column oven, a CBM‐20A system controller and a FRC‐10A fraction collector. LC–MS analysis was performed on a Waters Acquity UPLC with TQD mass detector (ESI). High‐resolution mass spectra (ESI) were recorded on an Orbitrap XL (Thermo Fisher Scientific).

**Synthesis of (2*R*)‐3‐(((2‐((*S*)‐2‐(6‐(2,5‐dioxo‐2,5‐dihydro‐1*H*‐pyrrol‐1‐yl)hexanamido)‐3‐(4‐(prop‐2‐yn‐1‐yloxy)phenyl)propanamido)ethoxy)(hydroxy)phosphoryl)oxy)propane‐1,2‐diyl dipalmitate (5)**: (*S*)‐2‐(6‐(2,5‐dioxo‐2,5‐dihydro‐1*H*‐pyrrol‐1‐yl)hexanamido)‐3‐(4‐(prop‐2‐yn‐1‐yloxy)phenyl)propanoic acid **4** (100 mg, 0.25 mmol, 3.5 equiv) was dissolved in freshly distilled THF (3 mL) in a 10 mL Schlenk flask under an argon atmosphere. *N*‐Hydroxysuccinimide (40 mg, 0.35 mmol, 4.8 equiv) and dicyclohexylcarbodiimide (65 mg, 0.3 mmol, 4.1 equiv) were added, and the mixture was stirred for 2 h until the starting material was fully consumed on TLC. The mixture was then filtered over Celite to remove the white precipitate and concentrated to about 1 mL in volume. While stirring the first solution, a second mixture of DPPE (50 mg, 0.07 mmol, 1 equiv) in chloroform (4 mL) was made using a few drops of 2,6‐lutidine to aid the dissolving process (repeated heating/sonication cycles were required). After cooling down the DPPE‐solution to room temperature, it was placed in a sonicating bath before the concentrated solution containing the O‐succinimide was added dropwise, making sure the solution did not precipitate. After addition, the clear solution was left to stir for 20 h. The mixture was then diluted to 10 mL using additional chloroform and washed with 0.1 m HCl (aq.) (10 mL), water (10 mL) and brine (10 mL). The organic layer was then dried over Na_2_SO_4_, filtered and dried in vacuo, to obtain the crude product as a yellow oil. Flash chromatography (silica, MeOH/CH_3_Cl/NH_3_ 5:95:0.1 to 20:80:0.1 in three steps) afforded the product as a clear oil that later turned into a glassy film (43 mg, 0.04 mmol, 57 %). ^1^H NMR (400 MHz, CDCl_3_): *δ*=7.13 (d, *J*=8.1 Hz, 2 H), 6.84 (d, *J*=8.2 Hz, 2 H), 6.69 (s, 2 H), 5.16 (m, 1 H), 4.82 (m, 1 H), 4.64 (d, *J*=2.4 Hz, 2 H), 4.33 (d, *J*=11.6 Hz, 1 H), 3.94 (m, 7 H), 3.39 (t, *J*=7.3 Hz, 2 H), 3.06 (m, 1 H), 2.83 (m, 1 H), 2.54 (t, *J*=2.4 Hz, 1 H), 2.23 (t, *J*=7.7 Hz, 4 H), 2.05 (m, 2 H), 1.53 (m, 4 H), 1.39 (m, 4 H), 1.24 (m, 48 H), 1.06 (m, 2 H), 0.86 ppm (m, 6 H); HRMS (ESI^−^): *m*/*z* calcd for C_59_H_95_N_3_O_13_P: 1084.661 [*M*−H]^−^, found 1084.662.

**General procedure for labeling with Sulfo‐Cy3 (Example tyrosine‐based linker without DPPE (4)) (6)**: Maleimidohexyl modified *O*‐propargyloxy‐l‐tyrosine carboxylic acid **4** (0.42 mg, 1 μmol, 1 equiv) and Na‐ascorbate (0.6 mg, 3 μmol, 3 equiv) were added to a stained (brown) HPLC vial (1.5 mL) equipped with a screw‐cap and solubilized using 50 μL TFE. Sulfo‐Cy3 Azide (0.83 mg, 1.33 μmol, 1.33 equiv) in 50 μL *t*BuOH/ddH_2_O 5:1 was then added. A mixture of CuSO_4_ (1.2 mg, 7.5 μmol) and THPTA (5.3 mg, 12.2 μmol) in 2.79 mL ddH_2_O was prepared simultaneously and 46 μL of this solution was added to the reaction vial. The mixture was stirred at RT in the dark overnight, then filtered using a 0.45 μm microfilter and purified using semi‐preparative HPLC (Waters Cortecs HILIC 2.7 μm 4.6×150 mm column, solvent A: 0.1 % FA in ACN, solvent B: 0.1 % FA in ddH_2_O, gradient: 5 % B to 45 % B over 30 min, flow: 0.5 mL min^−1^). Analytical HPLC (same conditions): *t*_R_=22 min (broad). The pure fractions were pooled and lyophilized and the product (**6**) was obtained as a purple solid (0.5 mg, 0.47 μmol, 47 %). HRMS (ESI^−^) *m*/*z* calcd for C_55_H_65_N_8_O_13_S_2_^−^: 1109.412 [*M*−2 H]^−^, found 1109.411.

**Synthesis of tyrosine‐based linker with DPPE labelled with Sulfo‐Cy3 (7)**: Maleimidohexyl (dipalmitoylglyceryl‐phosphatidyl)ethanolamide modified *O*‐propargyloxytyrosine **5** (1.1 mg, 1 μmol, 1 equiv) was treated according to the above general procedure. The mixture was filtered using a 0.45 μm microfilter and purified using semi‐preparative HPLC (XBridge C_18_ 5 μm 4.6×150 mm column, solvent A: 0.1 % NH_3_ in ACN, solvent B: 0.1 % NH_3_ in ddH_2_O, gradient: 60 % B to 5 % B over 40 min, flow: 0.5 mL min^−1^). The obtained fractions were analyzed using LC–MS (ESI^−^, Waters BEH C_18_ 1.7 μm 2.1×50 mm column, solvent A: 0.1 % NH_3_ in ddH_2_O, solvent B: 0.1 % NH_3_ in ACN, gradient: 90 % A to 50 % A over 8 min, to 5 % A in 1 min, total runtime: 15 min) *t*_R_=11.28 min, *m*/*z=*1782.9 [*M*−2 H]^−^. The pure fractions were pooled and lyophilized and the product (**7**) was obtained as a purple solid (0.43 mg, 0.24 μmol, 24 %). HRMS (ESI^−^) *m*/*z* calcd for C_92_H_136_N_9_O_20_PS_2_^2−^: 890.955 [*M*−3 H]^2−^, found 890.953.

**Synthesis of WALP‐Sulfo‐Cy3 hybrid using Sulfo‐Cy3 labelled tyrosine‐based linker without DPPE (8)**: WALP (1.5 mg, 0.48 μmol; ≈80 % purity) was placed in a Schlenck tube, which was brought under argon atmosphere; 250 μL of a 0.94 mm solution of Sulfo‐Cy3 labeled tyrosine‐based linker without DPPE (**6**) in TFE (0.24 μmol) was added, followed by 0.84 μL DIPEA and the mixture was stirred in the dark at RT overnight. 100 μL ddH_2_O/ACN 1:1 was added, and the mixture was filtered using a 0.45 μm microfilter. The clear filtrate was purified using semi‐preparative HPLC (XBridge C_18_ 5 μm 4.6×150 mm column, solvent A: 0.1 % NH_3_ in ACN, solvent B: 0.1 % NH_3_ in ddH_2_O, gradient: 60 % B to 5 % B over 40 min, flow: 0.5 mL min^−1^). The obtained fractions were analyzed using LC–MS (ESI^−^, Waters BEH C_18_ 1.7 μm 2.1×50 mm column, solvent A: 0.1 % NH_3_ in ddH_2_O, solvent B: 0.1 % NH_3_ in ACN, gradient: 90 % A to 50 % A over 8 min, to 5 % A in 1 min, total run time: 15 min); *t*_R_=7.55 min, *m*/*z=*1248.0 [*M*−4H]^3−^; HRMS (ESI^−^) *m*/*z* calcd for C_187_H_261_N_37_O_39_S_3_^2−^: 1872.438 [*M*−3 H]^2−^, found 1872.440; *m*/*z* calcd for C_187_H_260_N_37_O_39_S_3_^3−^: 1247.956 [*M*−4 H]^3−^, found 1247.960; *m*/*z* calcd for C_187_H_259_N_37_O_39_S_3_^4−^: 935.715 [*M*−5 H]^4−^, found 935.718.

**Synthesis of WALP‐Sulfo‐Cy3 hybrid using Sulfo‐Cy3 labelled tyrosine‐based linker with DPPE (9)**: WALP (0.8 mg, 0.24 μmol; ≈80 % purity) was placed in a Schlenck tube, which was brought under Ar atmosphere; 250 μL of a 0.48 mm solution of Sulfo‐Cy3 labeled tyrosine‐based linker with DPPE (**7**) in TFE (0.12 μmol) was added, followed by 0.42 μL DIPEA and the mixture was stirred in the dark at room temperature overnight. 100 μL ddH_2_O/ACN 1:1 was added and the mixture was filtered using a 0.45 μm microfilter. The clear filtrate was purified using semi‐preparative HPLC (XBridge C_8_ 5 μm 4.6×150 mm column, solvent A: 0.1 % NH_3_ in ACN, solvent B: 0.1 % NH_3_ in ddH_2_O, gradient: 60 % B to 5 % B over 40 min, flow: 0.5 mL min^−1^). The obtained fractions were analyzed using LC–MS (ESI^−^, Waters Protein BEH C_4_ 1.7 μm 2.1×150 mm column, solvent A: 0.1 % NH_3_ in ddH_2_O, solvent B: 0.1 % NH_3_ in ACN, gradient: 70 to 30 % A over 8 min, to 5 % A in 1 min, total run time: 20 min), *t*_R_=10.82 min, *m*/*z=*1472.7 [*M*−4 H]^3−^; HRMS (ESI^−^): *m*/*z* calcd for C_224_H_332_N_38_O_46_PS_3_^3−^: 1472.458 [*M*−4 H]^3−^, found 1472.457; *m*/*z* calcd for C_224_H_331_N_38_O_46_PS_3_^4−^: 1104.091 [*M*−5H]^4−^, found 1104.090.

**GUV formation**: GUVs composed of saturated lipids (DPPC, DSPC), double mono‐unsaturated lipids (DOPC, 16:1 PC), and cholesterol in a 4:3:3 molar ratio were formed by electroformation.^45^ For immobilization purposes 0.1 mol % of DOPE was added to each of the mixtures, as described previously.^51^ The labelled WALP peptide together with the L_d_ phase markers ATTO 655 DOPE, or DiD, were added to the lipid mixture in a 1:1000 molar ratio, unless stated otherwise. Atto 655 is a water‐soluble, zwitterionic fluorophore coupled to a lipid that is excited in the far red and spectrally distinct from Sulfo‐Cy3. Around 400 nmol of lipids, together with L_d_ marker and WALP were spotted on a conductive indium tin oxide (ITO)‐coated glass plate. After removal of the solvents under vacuum, GUVs were formed in 200 mm sucrose on the Vesicle Prep Pro (Nanion technologies) with a voltage of 1.1 V at 10 Hz for 1 h at 50 °C.

**Immobilization of GUVs**: GUVs were immobilized on slides modified with APTES‐glutaraldehyde as described previously.^51^ In short, coverslips were cleaned in KOH and plasma cleaned. They were then modified in 2 % APTES solution for 10 seconds and stored in vacuum until the day of the experiment but always within 48 h. Prior to the experiment, slides were incubated with 5 % glutaraldehyde for 30 min, after which the glutaraldehyde was washed away with ddH_2_O. Subsequently, the GUVs, diluted 10 times in 100 mm NaCl, were placed on the slide.

**Imaging**: GUVs were imaged on the Zeiss LSM 710 confocal microscope with a 40x C‐Apochromat Corr M27 with NA 1.2 water immersion objective. ATTO 655 DOPE and DiD were excited with a 633 nm HeNe laser; the Sulfo‐Cy3 coupled to the WALP peptide was excited with a 543 nm HeNe laser. Z‐stack images of immobilized GUVs were taken. The images of both channels were taken separately to avoid cross talk.

**Image analysis**: The GUVs were automatically identified by detecting circles in the image. The detection of circles was done using a circle Hough transform^82^ for a range of radii, resulting in a stack of images; one image for each radius. We then created a maximum intensity projection where each pixel contains the maximum value over all images in the stack at the particular pixel location. On this projection we detect peaks by repeatedly finding the brightest pixel, which gives us the centers of the detected GUVs. The radius of each GUV corresponds to the radius on which the maximum pixel value was found when we created the maximum intensity projection (Figure S2).

After detection of the circles on the image, the non‐phase‐separating GUVs were filtered out. We calculated the Pearson's correlation coefficient for the fluorescent profile of the L_d_ marker relative to the same profile that was smoothened. Smoothening was done with a moving average filter. A high correlation indicates two separate phases, while low correlation is consistent with a single‐phase vesicle. We used the correlation of 0.9 as a cut off for phase‐separating vesicles. After the automatic detection all selections were manually inspected and false positives were removed. We used the smoothened profile of the L_d_ marker to classify each intensity value as being either in the L_d_ or L_o_ phase based. Next, the correlation between the L_d_ marker and target construct, and the ratio of the mean intensity of the fluorescence in the L_o_ and L_d_ phases, were calculated on the non‐smoothened profiles (an overview of the data analysis is presented in Figure S3).

**FRAP experiments**: FRAP measurements were performed by imaging a small area of the membrane of the GUVs to achieve an acquisition time below 40 ms. A spot with a diameter of 1 μm was bleached at high laser intensity, after which the attenuated laser was used to record images every 40 ms for 6 s; the pre‐bleaching fluorescence was obtained from five images prior to the bleach. The halftime of recovery and lateral diffusion coefficients were calculated as described previously,^83^ which is based on work of Axelrod and colleagues.^84^

## Conflict of interest

*The authors declare no conflict of interest*.

## Supporting information

As a service to our authors and readers, this journal provides supporting information supplied by the authors. Such materials are peer reviewed and may be re‐organized for online delivery, but are not copy‐edited or typeset. Technical support issues arising from supporting information (other than missing files) should be addressed to the authors.

Supplementary
