# Supplementary material for: A Trifunctional Linker for Palmitoylation and Peptide and Protein Localization in Biological Membranes
Source: Chembiochem. 2020 Jan 10;21(9):1320–8. doi: 10.1002/cbic.201900655 (PMC7317724; doi:10.1002/cbic.201900655)
Supplement: Supplementary file 1 — Supplementary [file CBIC-21-1320-s001.pdf]

CHEMBIOCHEM

Supporting Information

A Trifunctional Linker for Palmitoylation and Peptide and Protein Localization in Biological Membranes

Łukasz Syga,[b] Reinder H. de Vries,[a] Hugo van Oosterhout,[a] Rianne Bartelds,[b] Arnold J. Boersma,[c] Gerard Roelfes,*[a] and Bert Poolman*[b]

cbic_201900655_sm_miscellaneous_information.pdf

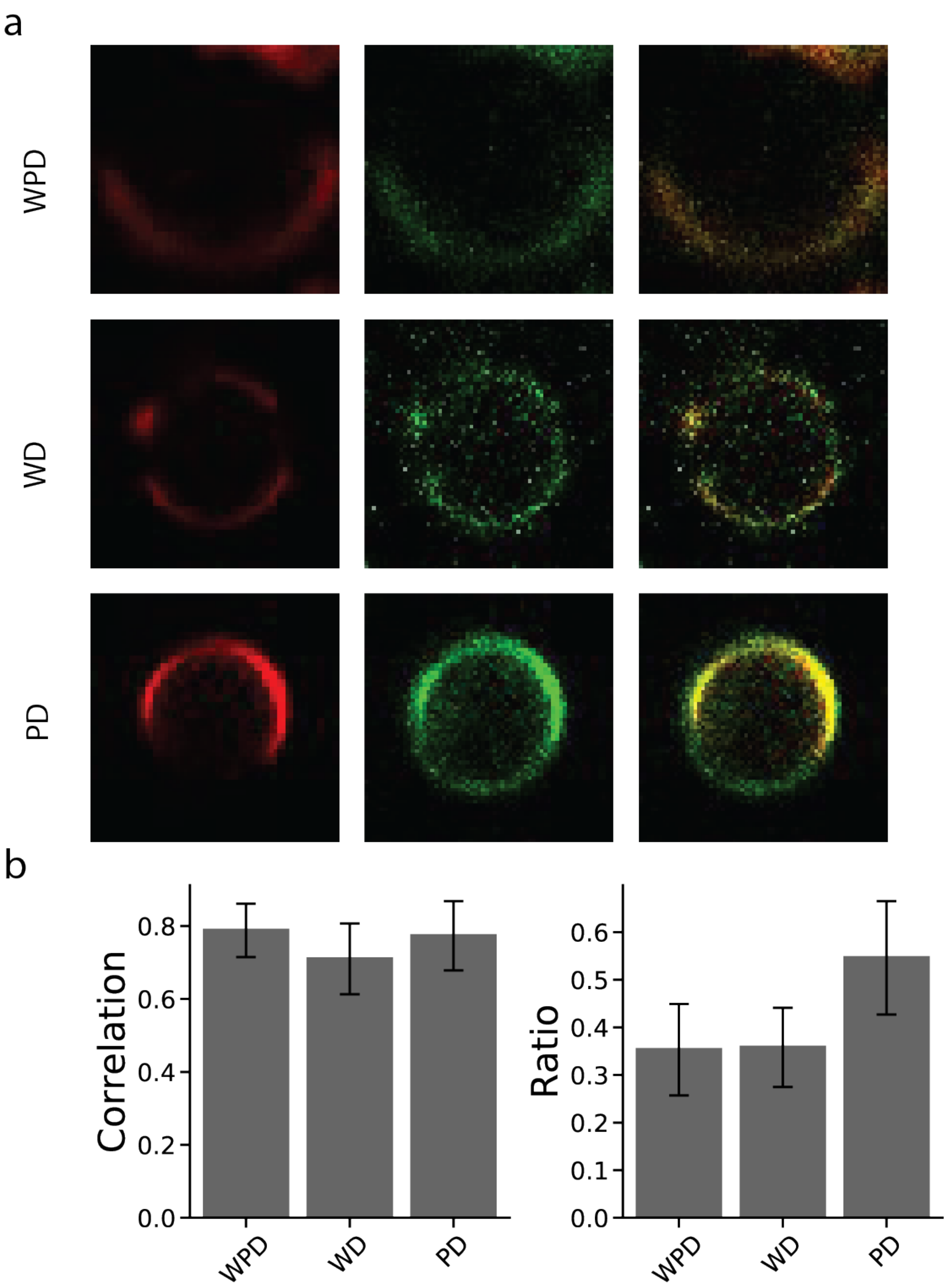

Supplementary Figure 1. Partitioning of the constructs within the membrane domains of phase-separating GUVs. Compared to the experiments presented in Figure 1 a 100-fold lower concentration of WPD, WD or PD was used, resulting in 1:100,000 protein to lipid ratios. Panel a shows images of GUVs with the SulphoCy3-labeled constructs in green and the L_d marker in red (Atto655). Panel b shows the Pearson correlation between construct and marker (left panel), and the ratio of the molecules in the L_o and L_d phase of the membrane (right panel). Error bars represent standard deviation of the sample.

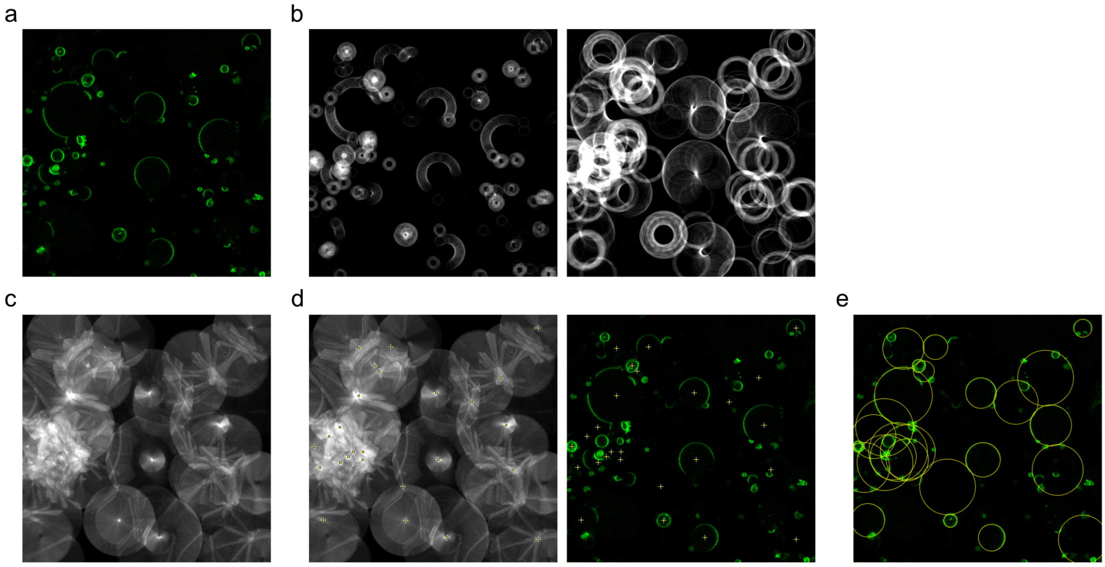

Supplementary figure 2. Automatic detection of GUVs. Panel a shows an original image on which we detect GUVs. Panels b show two examples of the circle Hough transformation for circles with different radii. Panel c shows the projection of the maximal values for all radii. Panels d show the detected centers of the GUVs on the projection (left) and the original image (right). Panel e shows the detected GUVs, which were later manually inspected and false positives were removed.

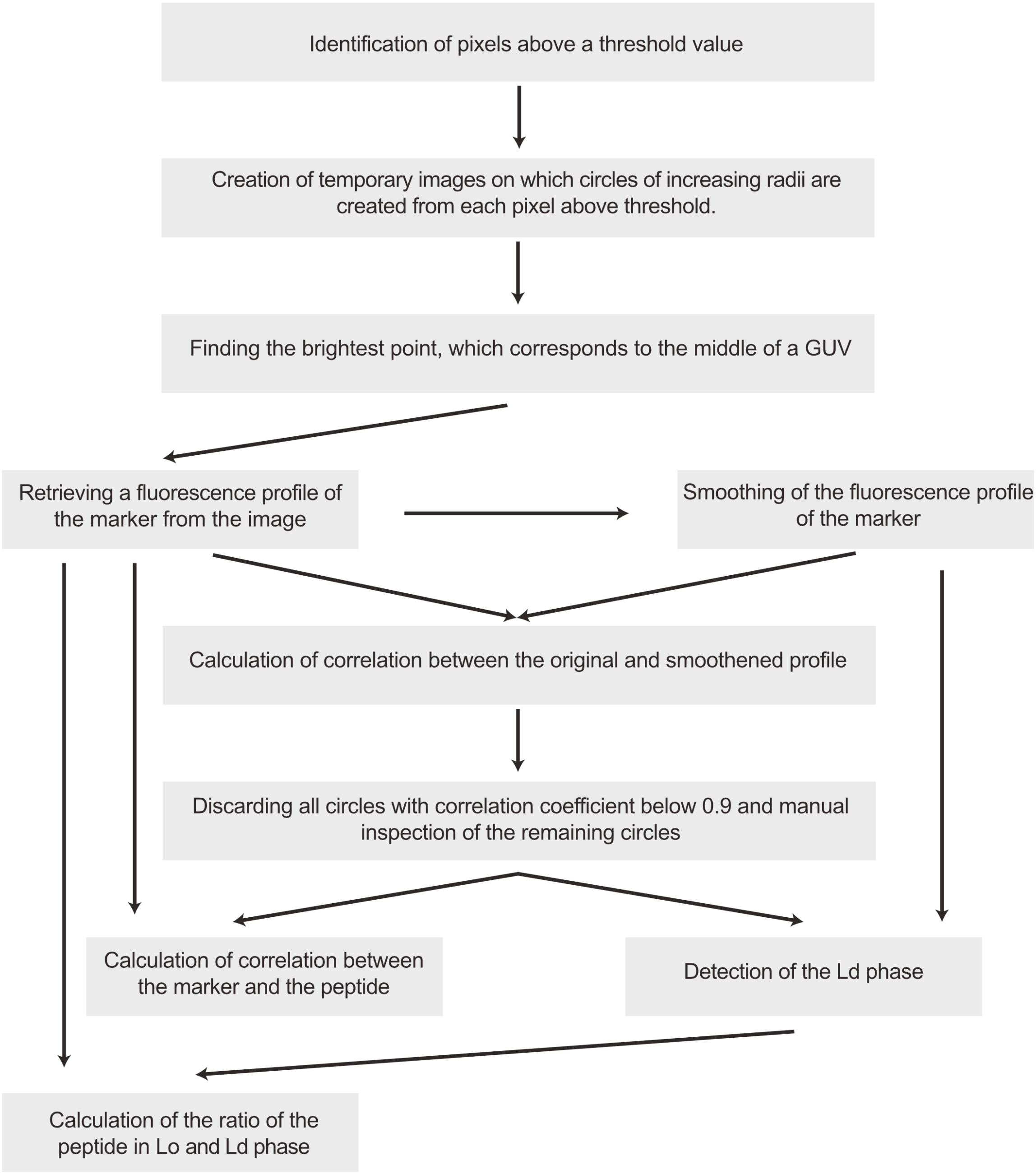

Supplementary Figure 3. Flowchart presenting the analysis of the localization data.

Supplementary Materials and methods

General remarks. All solvents and reagents were purchased from commercial suppliers and used without further purification. WALP (GCGWW(LA)$_8$LWWA) was purchased from Bachem. Sulfo-Cyanine3 azide (Sulfo-Cy3) was purchased from Lumiprobe. Reactions were monitored by TLC Silica 60 (Merck Millipore), examined under UV (365 nm and 254 nm), and stained by $KMnO_4$, ninhydrin, vanillin or H_2SO_4 in MeOH (1%). Flash chromatography was performed on Silica gel 60 (0.040-0.063 mm) from Merck Millipore. ^{1}H NMR spectra were recorded at 300 or 400 MHz and ^{13}C NMR spectra were recorded at 75 MHz. The chemical shifts are reported in ppm relative to the residual solvent peak ($CDCl_3$ at δH = 7.26 ppm, δC = 77.16 ppm). Yields of the dye constructs were based on UV absorption at 548 nm and the molar absorptivity coefficient of Sulfo-Cy3 azide (ε = 162000 M^{-1} cm^{-1} at 548 nm), not compensating for the presence of lipid or linker (assumed to not absorb in that region). LC-MS analysis was performed on a Waters Acquity UPLC with TQD mass detector (ESI). High-resolution mass spectra (ESI) were recorded on an Orbitrap XL (Thermo Fisher Scientific).

Synthesis of N-Boc-O-propargyl-L-Tyrosine methyl ester (1). Boc-L-tyrosine methyl ester (6 g, 20 mmol, 1 equiv.) was dissolved in 100 mL dry DMF , K_2CO_3 (11 g, 80 mmol, 4 equiv.) was added and the suspension was stirred vigorously. Propargylbromide (80% in toluene, 5.2 mL, 40 mmol, 2 equiv.) was added and the resulting mixture stirred for 6h. The solution was concentrated to approx. 15 mL *in vacuo* and diluted with 100 mL EtOAc. The organic phase was then washed with 100 mL ammonia (sat. aq.), 100 mL water, 3x100 mL 3M LiCl (aq.) and 100 mL brine, dried over Na_2SO_4, filtered and concentrated *in vacuo.* Flash chromatography of the crude (silica, 10% EtOAc in heptane) afforded the product as a white solid (5.84 g, 17.5 mmol, 88 %). ^{1}H NMR (300 MHz, $CDCl_3$) δ 7.05 (d, J = 8.3 Hz, 2H), 6.90 (d, J = 8.6 Hz, 2H), 4.96 (d, J = 8.4 Hz, 1H), 4.67 (d, J = 2.4 Hz, 2H), 4.60 – 4.46 (m, 1H), 3.71 (s, 3H), 3.17 – 2.83 (m, 2H), 2.51 (t, J = 2.3 Hz, 1H), 1.42 (s, 9H); ^{13}C NMR (75 MHz, $CDCl_3$) δ 172.5, 156.8, 155.2, 130.5, 129.1, 115.1, 80.0, 78.7, 75.6, 56.0, 54.6, 52.3, 37.6, 28.4.

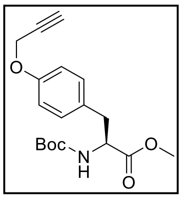

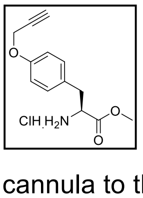

Synthesis of O-propargyl-L-Tyrosine methyl ester HCl salt (2). N-Boc-O-propargyl-L-Tyrosine methyl ester **1** (5.84 g, 17.5 mmol, 1 equiv.) was dissolved in 50 mL anhydrous MeOH under a nitrogen atmosphere. Anhydrous HCl in MeOH was generated by slowly adding acetyl chloride (2.5 mL, 40 mmol, 2.4 equiv.) to 10 mL dry methanol cooled to 0°C. Upon completion of the acetyl chloride addition, the HCl solution was added via cannula to the first solution and left to stir for 30 min. The mixture was then concentrated *in vacuo*, and triturated with $CHCl_3$ (3 x 50 mL), to afford the product as a white fluffy powder (4.05 g, 15 mmol, 88 %), which was used in the next step without purification.

Synthesis of methyl (*S*)-2-(6-(2,5-dioxo-2,5-dihydro-1H-pyrrol-1-yl)hexanamido)-3-(4-(prop-2-yn-1-yloxy)phenyl)propanoate (3). O-propargyl-L-Tyrosine methyl ester HCl salt **2** (1.42 g, 5.25 mmol, 1.1 equiv.) and 6-maleimidohexanoic acid (1.06 g, 5 mmol, 1 equiv.) were dissolved in 50 mL CH_2Cl_2. At 0 °C, triethylamine (0.84 mL, 6 mmol, 1.2 equiv.) was added, followed by diisopropylcarbodiimide (0.95 mL, 6 mmol, 1.5 equiv.) and a single crystal of N,N-dimethylaminopyridine After 30 min, the reaction was allowed to warm to room temperature, after which the reaction was stirred for an additional 3 h, until full consumption of the starting material was observed by TLC. The mixture was filtered over a sintered glass filter (P4) and subsequently diluted with 50 mL CH_2Cl_2 washed with 100 mL 1M HCl (aq.), 100 mL $NaHCO_3$ (sat. aq.) and 100 mL brine. The organic phase was then dried over Na_2SO_4, filtered and concentrated *in vacuo*. Flash Chromatography (silica, 0-5 % EtOAc in Et_2O) afforded the product as a clear oil (777 mg, 1.82 mmol, 36 %). ^{1}H NMR (300 MHz, $CDCl_3$) δ 7.01 (d, J = 8.5 Hz, 2H), 6.90 (d, J = 8.7 Hz, 2H), 6.67 (s, 2H), 5.85 (d, J = 7.8 Hz, 1H), 4.84 (dt, J = 7.6, 5.6 Hz, 1H), 4.66 (d, J = 2.4 Hz, 2H), 3.72 (s, 3H), 3.49 (t, J = 7.3 Hz, 2H), 3.15 – 2.96 (m, 2H), 2.51 (t, J = 2.3 Hz, 1H), 2.15 (t, J = 7.6 Hz, 2H), 1.68 – 1.48 (m, 4H), 1.37 – 1.18 (m, 2H); ^{13}C NMR (75 MHz, $CDCl_3$) δ 172.3, 171.0, 156.9, 134.2, 130.4, 129.0, 115.1, 78.7, 75.7, 56.0, 53.1, 52.4, 37.7, 37.2, 36.3, 28.4, 26.4, 25.0 (1 carbonyl C missing due to overlap).

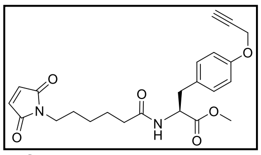

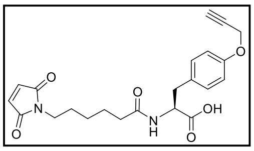

Synthesis of (*S*)-2-(6-(2,5-dioxo-2,5-dihydro-1H-pyrrol-1-yl)hexanamido)-3-(4-(prop-2-yn-1-yloxy)phenyl)propanoic acid (4). Methyl (*S*)-2-(6-(2,5-dioxo-2,5-dihydro-1H-pyrrol-1-yl)hexanamido)-3-(4-(prop-2-yn-1-yloxy)phenyl)propanoate **3** (430 mg, 1 mmol, 1 equiv.) was dissolved in 1,2-dichloroethane (5 mL) in a sealed tube. Me_3SnOH (200 mg, 1.1 mmol, 1.1 equiv.) was added and the solution was heated under reflux for 4 h, until full consumption of the starting material. The solution was then concentrated *in vacuo* and redissolved in 50 mL EtOAc. The organic phase was washed with 3x50 mL 1M HCl (aq.), dried over Na_2SO_4 and concentrated. Flash chromatography (silica, 0-20 % EtOH in CH_2Cl_2) afforded the product as a clear oil that solidified to a white solid over the course of several days (353 mg, 0.86 mmol, 86 %). ^{1}H NMR (300 MHz, $CDCl_3$) δ 7.08 (d, J = 8.3 Hz, 2H), 6.90 (d, J = 8.2 Hz, 2H), 6.67 (s, 2H), 6.11 (d, J = 7.6 Hz, 1H), 4.83 (q, J = 6.4 Hz, 1H), 4.65 (d, J = 2.2 Hz, 2H), 3.47 (t, J = 7.2 Hz, 2H), 3.26 – 2.99 (m, 2H), 2.52 (t, J = 2.3 Hz, 1H), 2.18 (t, J = 7.6 Hz, 2H), 1.71 – 1.44 (m, 4H), 1.32 – 1.19 (m, 2H). ^{13}C NMR (75 MHz, $CDCl_3$) δ 174.5, 173.6, 171.1, 156.9, 134.2, 130.5, 128.9, 115.2, 78.7, 75.7, 55.9, 53.3, 37.7, 36.5, 36.2, 28.3, 26.2, 25.0.

Labelling of WALP27 with AlexaFluor 488. WALP27 was labelled directly with AlexaFluor 488 maleimide by following a procedure by Killian *et al.*[1] 0.5 mg of WALP27 was dissolved in 200 µL 2,2,2-trifluoroethanol. 10 µL H_2O was added and the mixture was degassed by bubbling N_2. 2 µL triethylamine and 2.6 equivalents of AlexaFluor 488 C5 maleimide in MeOH were added subsequently, and the mixture was stirred under N_2 atmosphere in the dark at 4 °C for 3 days. 10 mL cold (-20 °C) methyl *tert*-butyl ether/hexane 1:1 was added to precipitate the peptide, which was then collected by centrifugation (5 min. at 5000 rpm). The supernatant was decanted and the previous step was repeated once more to further purify the peptide. TLC (SiO_2, $CHCl_3$/MeOH/H_2O 65:25:4) was used to confirm successful labelling of the peptide.

1. Holt, A., Koehorst, R. B. M., Rutters-Meijneke, T., Gelb, M. H., Rijkers, D. T. S., Hemminga, M. A. & Killian, J. A. Tilt and rotation angles of a transmembrane model peptide as studied by fluorescence spectroscopy. *Biophys. J.* **97,** 2258–2266 (2009).

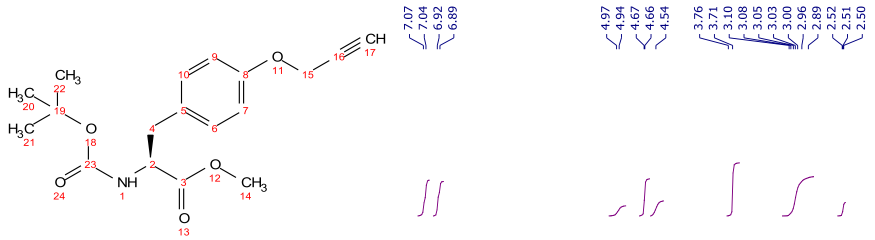

^{1}H NMR (300 MHz, Chloroform-*d*) δ 7.05 (d, *J* = 8.3 Hz, 2H), 6.90 (d, *J* = 8.6 Hz, 2H), 4.96 (d, *J* = 8.4 Hz, 1H), 4.67 (d, *J* = 2.4 Hz, 2H), 4.60 – 4.46 (m, 1H), 3.71 (s, 3H), 3.17 – 2.83 (m, 2H), 2.51 (t, *J* = 2.3 Hz, 1H), 1.42 (s, 9H).

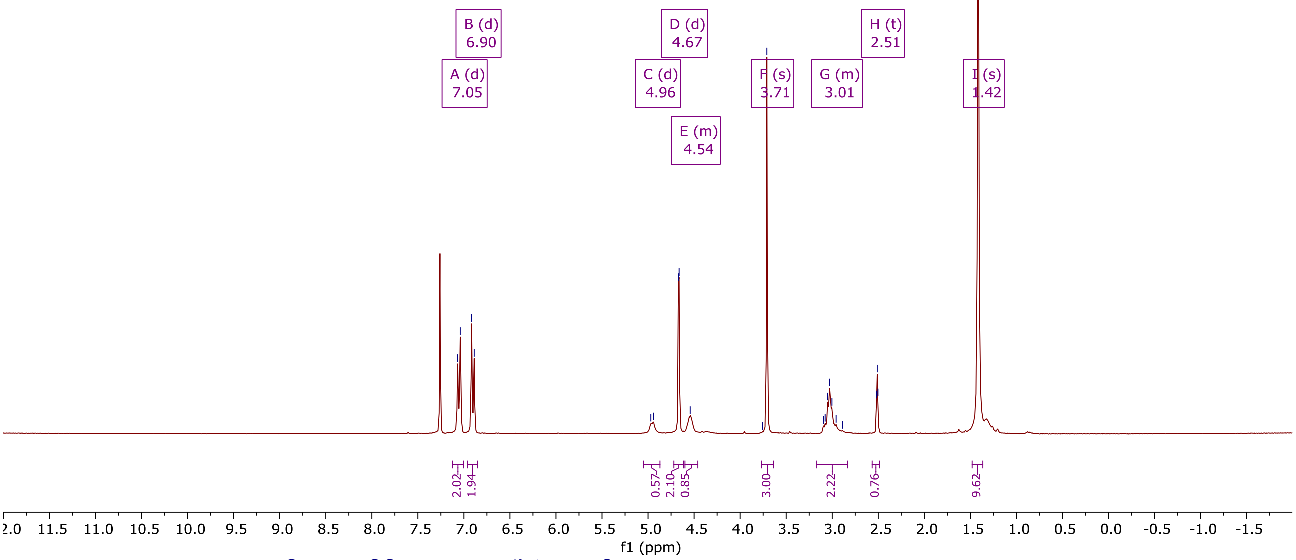

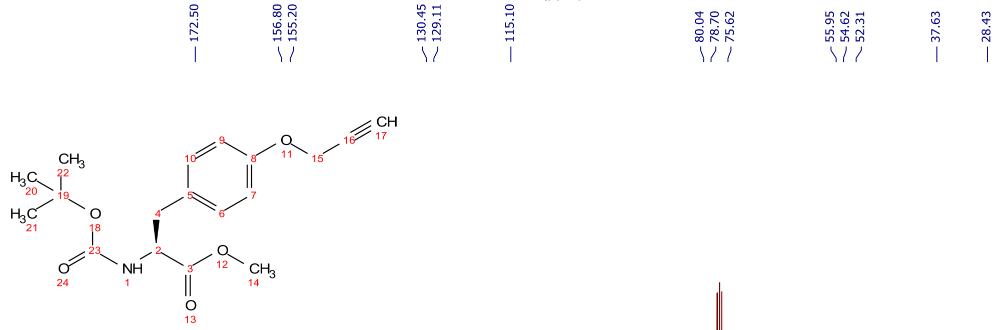

^{13}C NMR (75 MHz, $cdcl_3$) δ 172.50, 156.80, 155.20, 130.45, 129.11, 115.10, 80.04, 78.70, 75.62, 55.95, 54.62, 52.31, 37.63, 28.43.

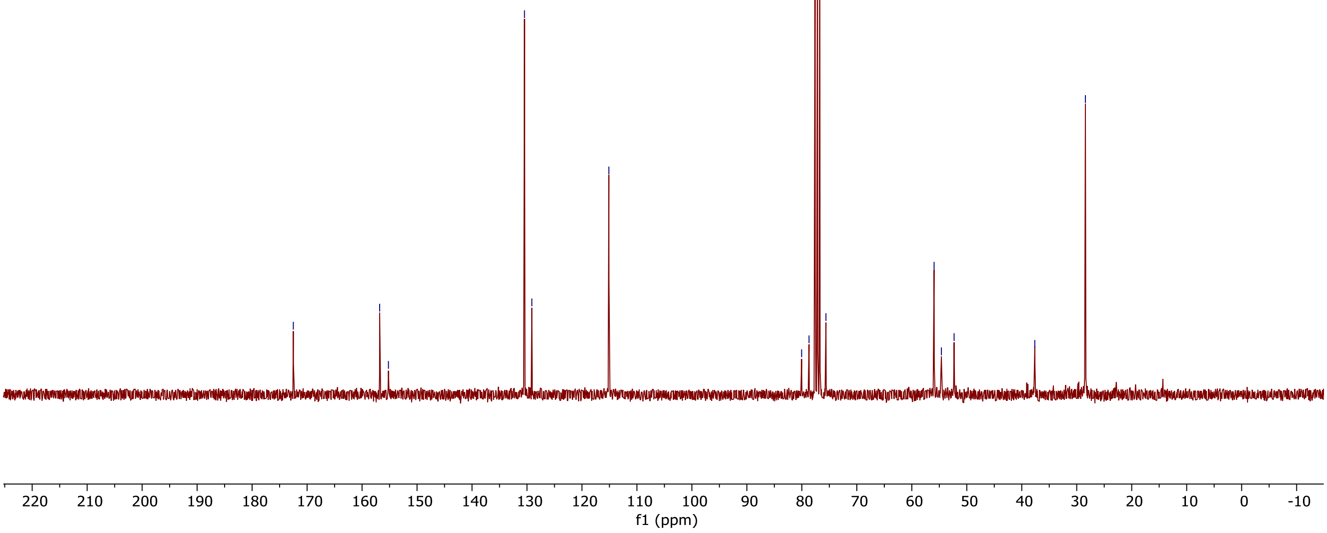

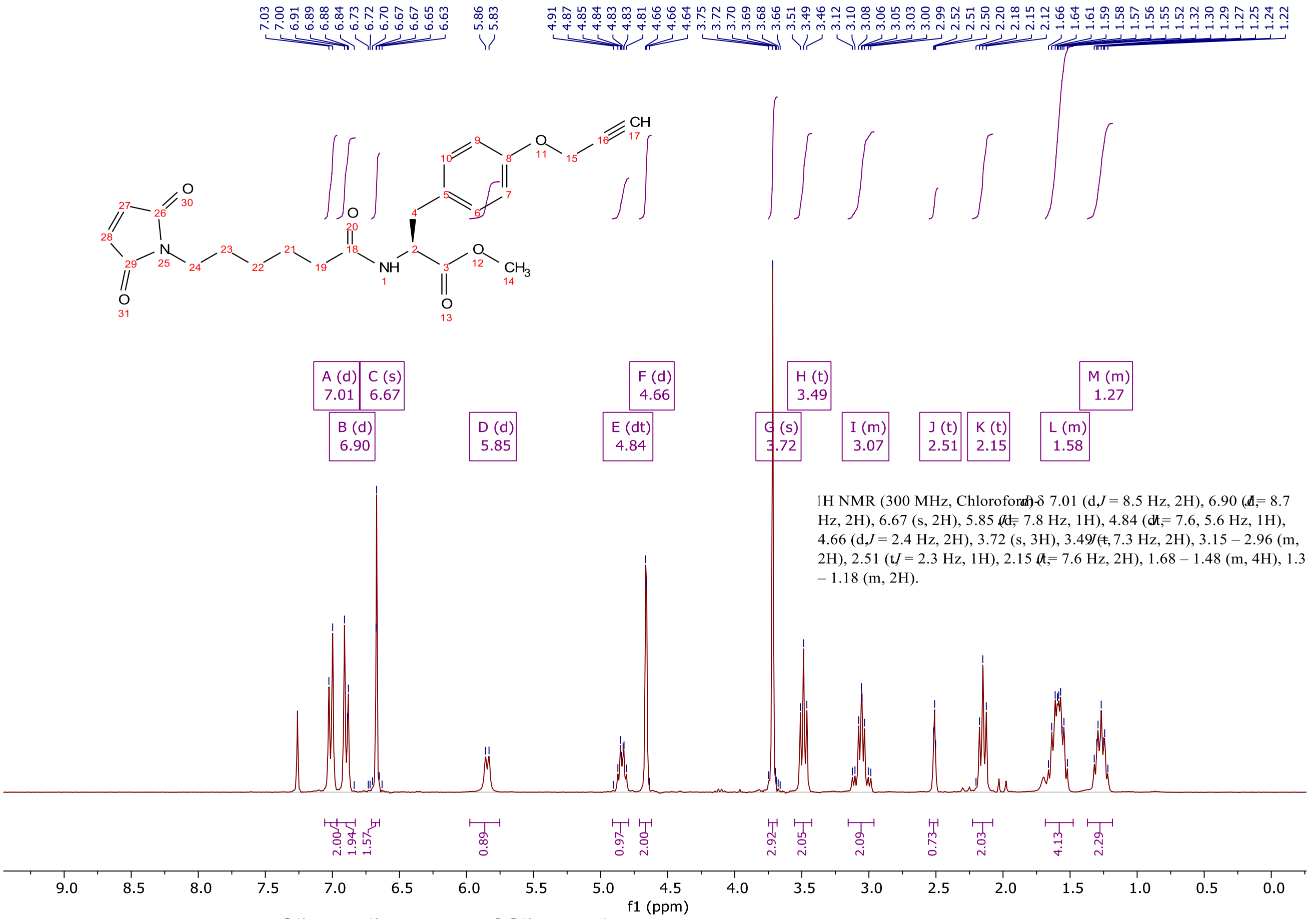

1H NMR (300 MHz, Chloroform-*d*) δ 7.01 (d, *J* = 8.5 Hz, 2H), 6.90 (d, *J* = 8.7 Hz, 2H), 6.67 (s, 2H), 5.85 (d, *J* = 7.8 Hz, 1H), 4.84 (dt, *J* = 7.6, 5.6 Hz, 1H), 4.66 (d, *J* = 2.4 Hz, 2H), 3.72 (s, 3H), 3.49 (t, *J* = 7.3 Hz, 2H), 3.15 – 2.96 (m, 2H), 2.51 (t, *J* = 2.3 Hz, 1H), 2.15 (t, *J* = 7.6 Hz, 2H), 1.68 – 1.48 (m, 4H), 1.3 – 1.18 (m, 2H).

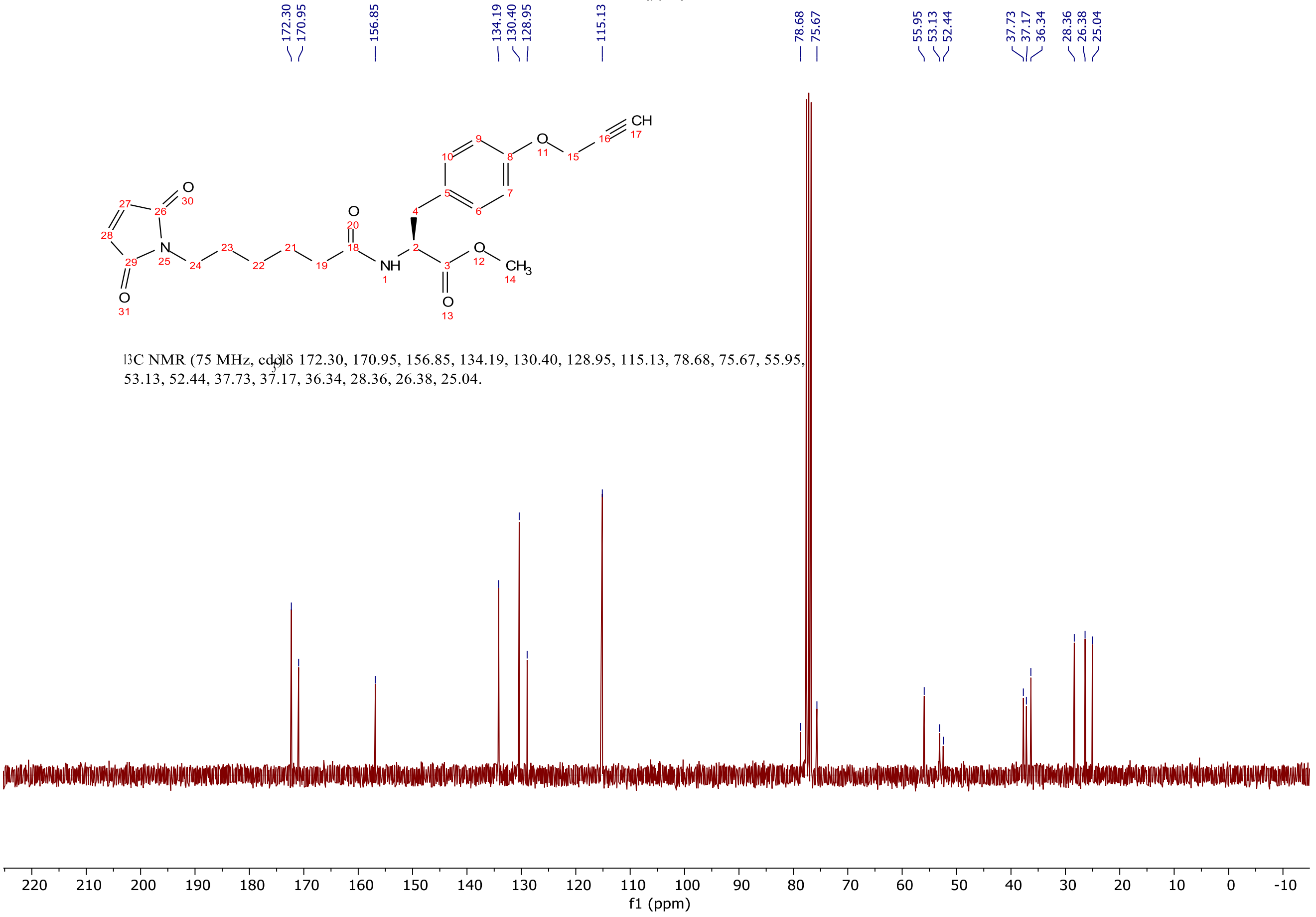

13C NMR (75 MHz, $cdcl_3$) δ 172.30, 170.95, 156.85, 134.19, 130.40, 128.95, 115.13, 78.68, 75.67, 55.95, 53.13, 52.44, 37.73, 37.17, 36.34, 28.36, 26.38, 25.04.

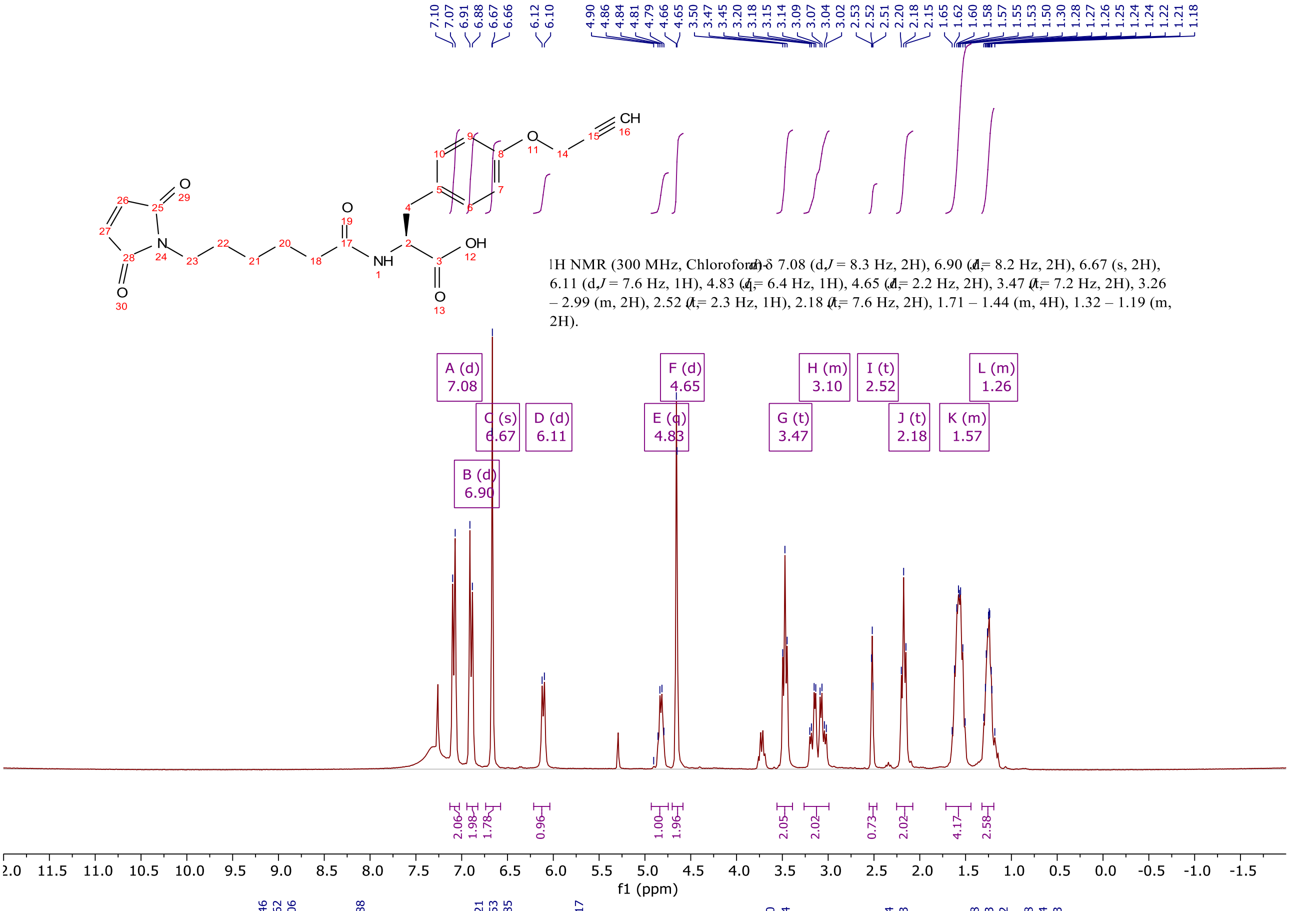

1H NMR (300 MHz, Chloroform-*d*) δ 7.08 (d, *J* = 8.3 Hz, 2H), 6.90 (d, *J* = 8.2 Hz, 2H), 6.67 (s, 2H), 6.11 (d, *J* = 7.6 Hz, 1H), 4.83 (q, *J* = 6.4 Hz, 1H), 4.65 (d, *J* = 2.2 Hz, 2H), 3.47 (t, *J* = 7.2 Hz, 2H), 3.26 – 2.99 (m, 2H), 2.52 (t, *J* = 2.3 Hz, 1H), 2.18 (t, *J* = 7.6 Hz, 2H), 1.71 – 1.44 (m, 4H), 1.32 – 1.19 (m, 2H).

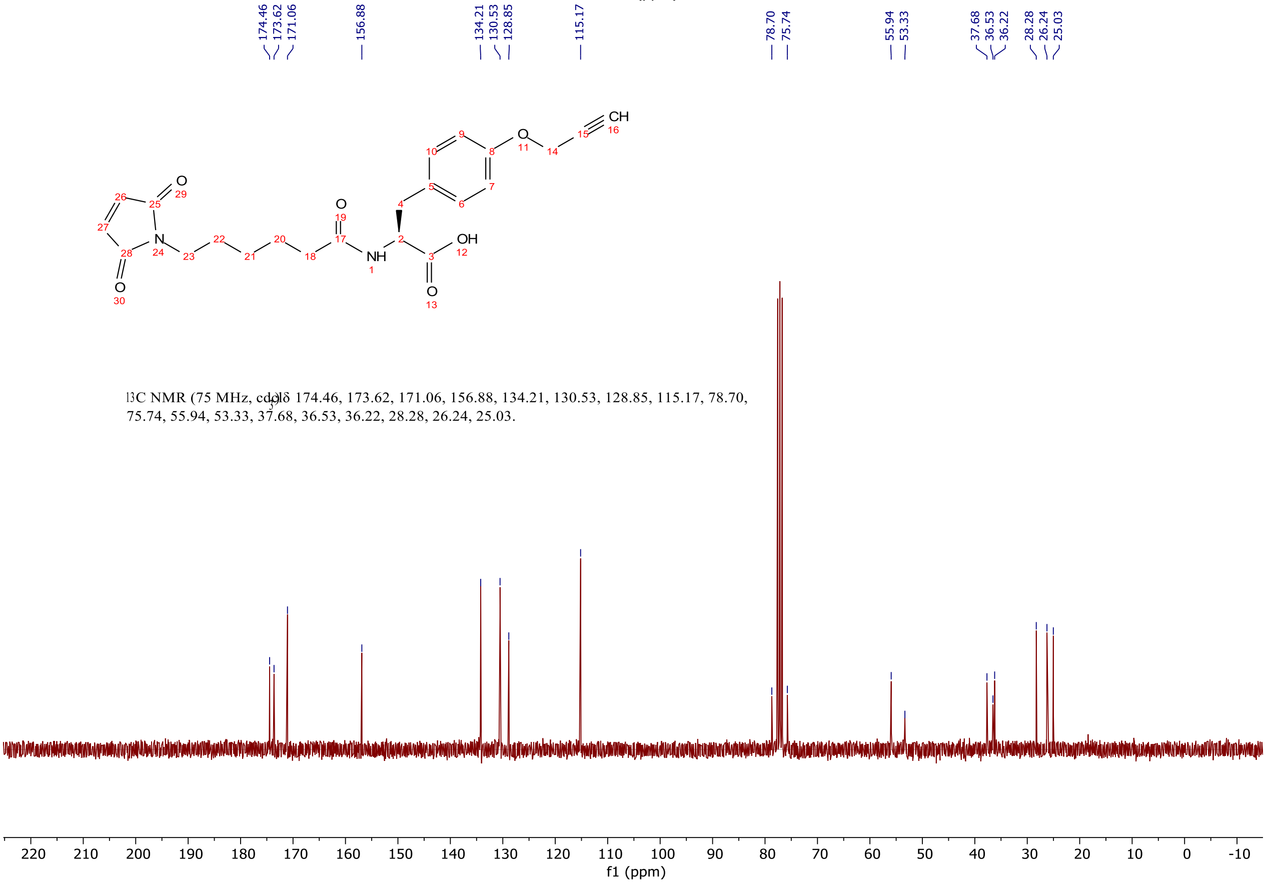

13C NMR (75 MHz, cdcl3) δ 174.46, 173.62, 171.06, 156.88, 134.21, 130.53, 128.85, 115.17, 78.70, 75.74, 55.94, 53.33, 37.68, 36.53, 36.22, 28.28, 26.24, 25.03.

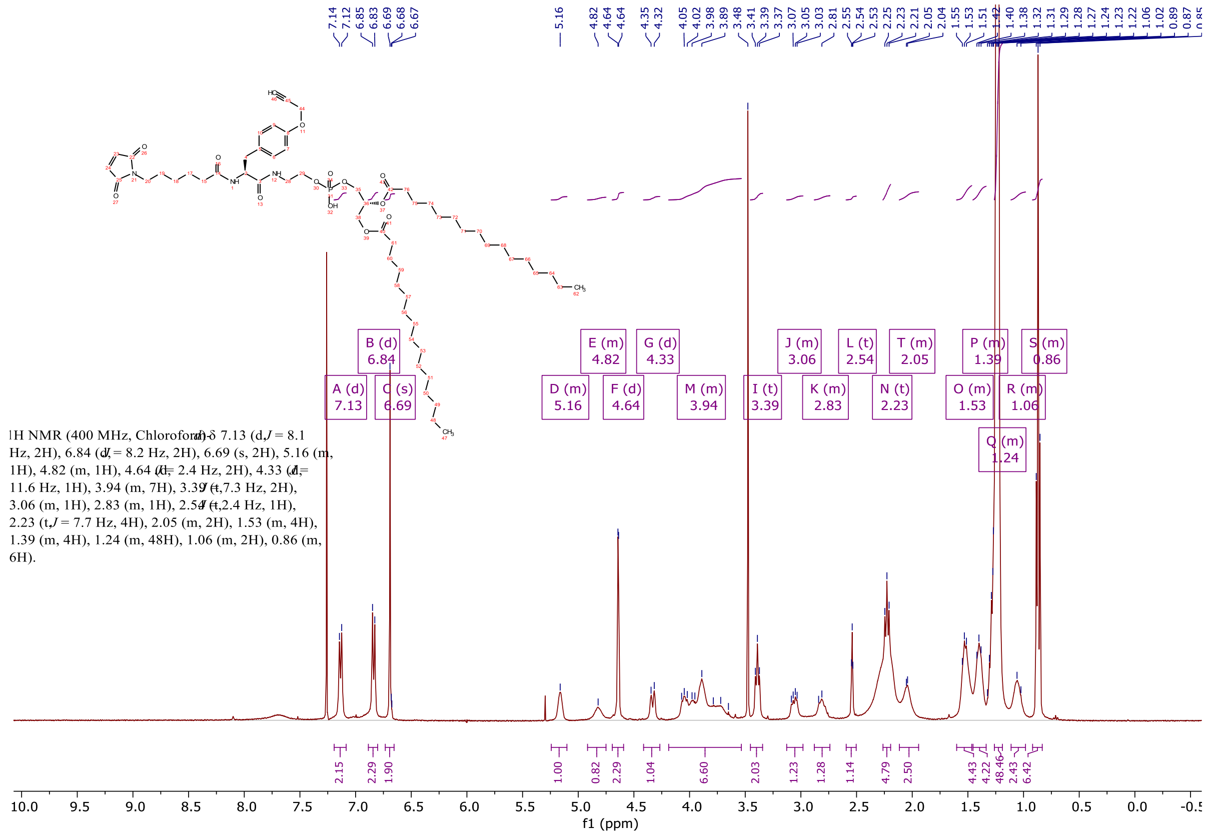
1H NMR (400 MHz, Chloroform-d) δ 7.13 (d, J = 8.1 Hz, 2H), 6.84 (d, J = 8.2 Hz, 2H), 6.69 (s, 2H), 5.16 (m, 1H), 4.82 (m, 1H), 4.64 (d, J = 2.4 Hz, 2H), 4.33 (d, J = 11.6 Hz, 1H), 3.94 (m, 7H), 3.39 (t, J = 7.3 Hz, 2H), 3.06 (m, 1H), 2.83 (m, 1H), 2.54 (t, J = 2.4 Hz, 1H), 2.23 (t, J = 7.7 Hz, 4H), 2.05 (m, 2H), 1.53 (m, 4H), 1.39 (m, 4H), 1.24 (m, 48H), 1.06 (m, 2H), 0.86 (m, 6H).
A (d) 7.13
B (d) 6.84
C (s) 6.69
D (m) 5.16
E (m) 4.82
F (d) 4.64
G (d) 4.33
M (m) 3.94
I (t) 3.39
J (m) 3.06
K (m) 2.83
L (t) 2.54
N (t) 2.23
T (m) 2.05
O (m) 1.53
P (m) 1.39
Q (m) 1.24
R (m) 1.06
S (m) 0.86
f1 (ppm)

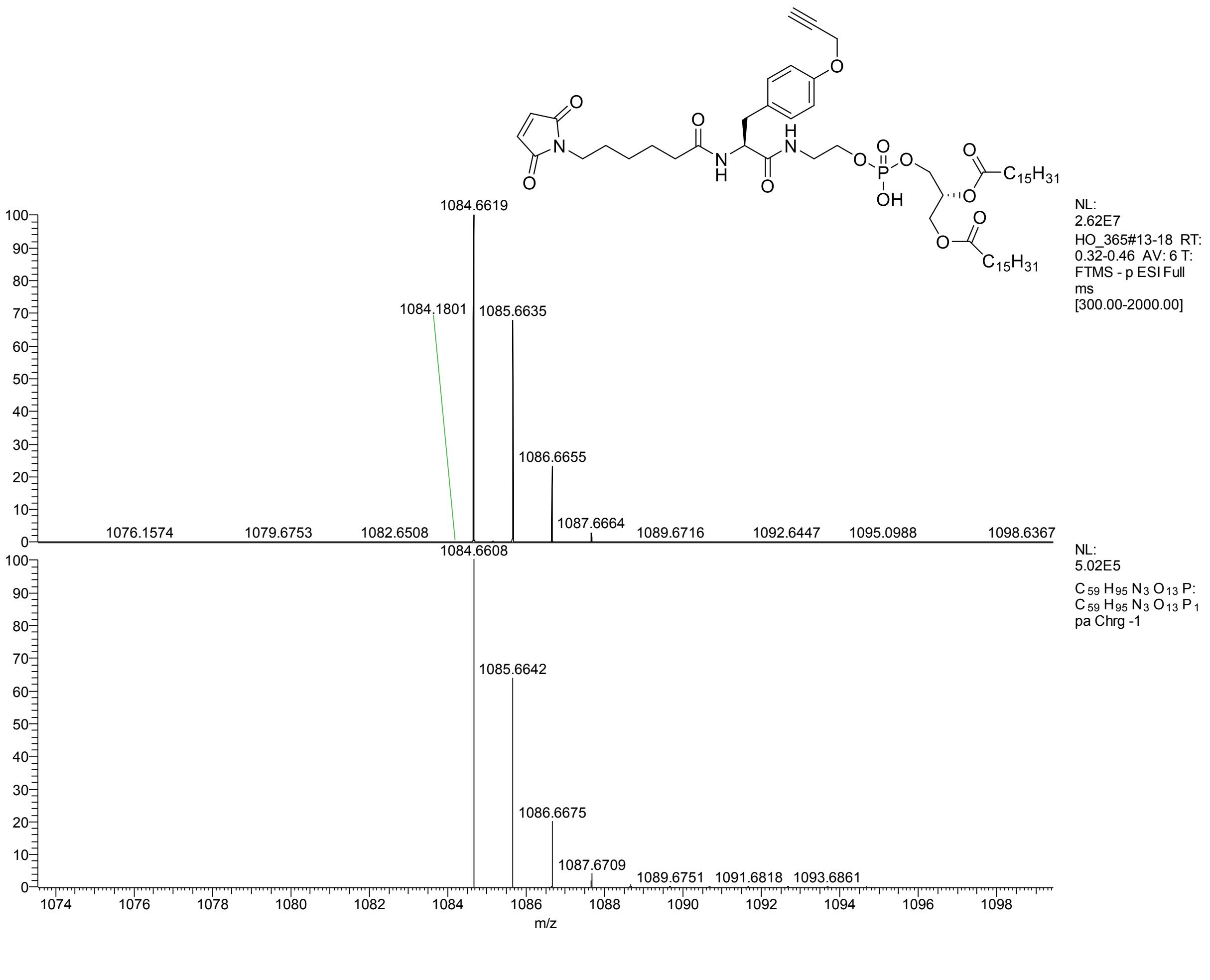
NL: 2.62E7
HO_365#13-18 RT: 0.32-0.46 AV: 6 T: FTMS - p ESI Full ms [300.00-2000.00]
1084.6619
1084.1801
1085.6635
1086.6655
1087.6664
NL: 5.02E5
C 59 H 95 N 3 O 13 P: C 59 H 95 N 3 O 13 P 1 pa Chrg -1
1084.6608
1085.6642
1086.6675
1087.6709
m/z

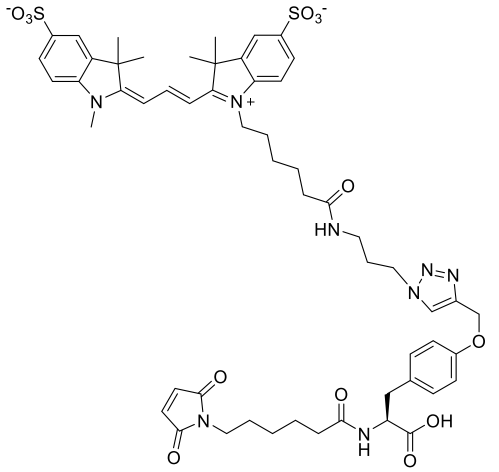

<Chromatogram>

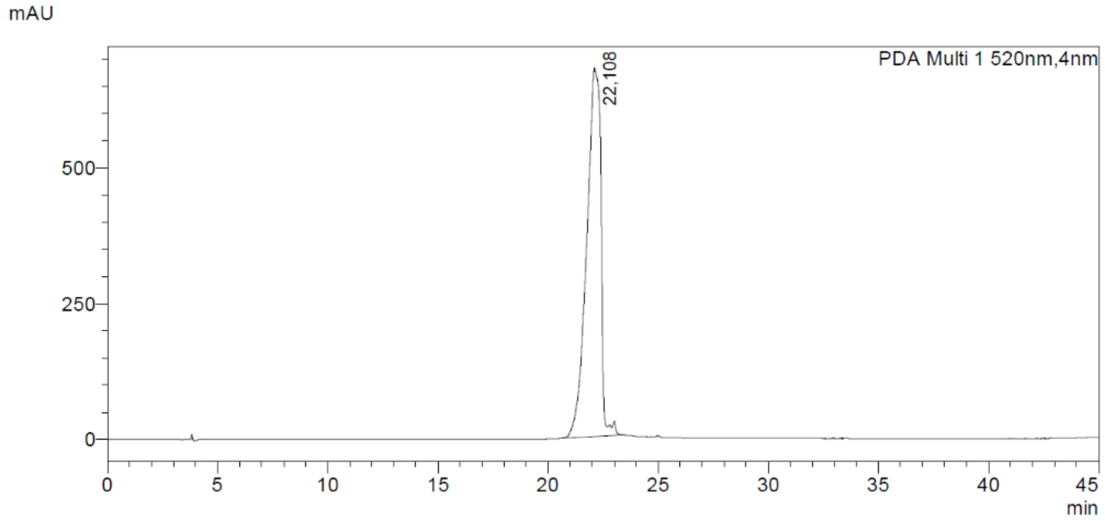

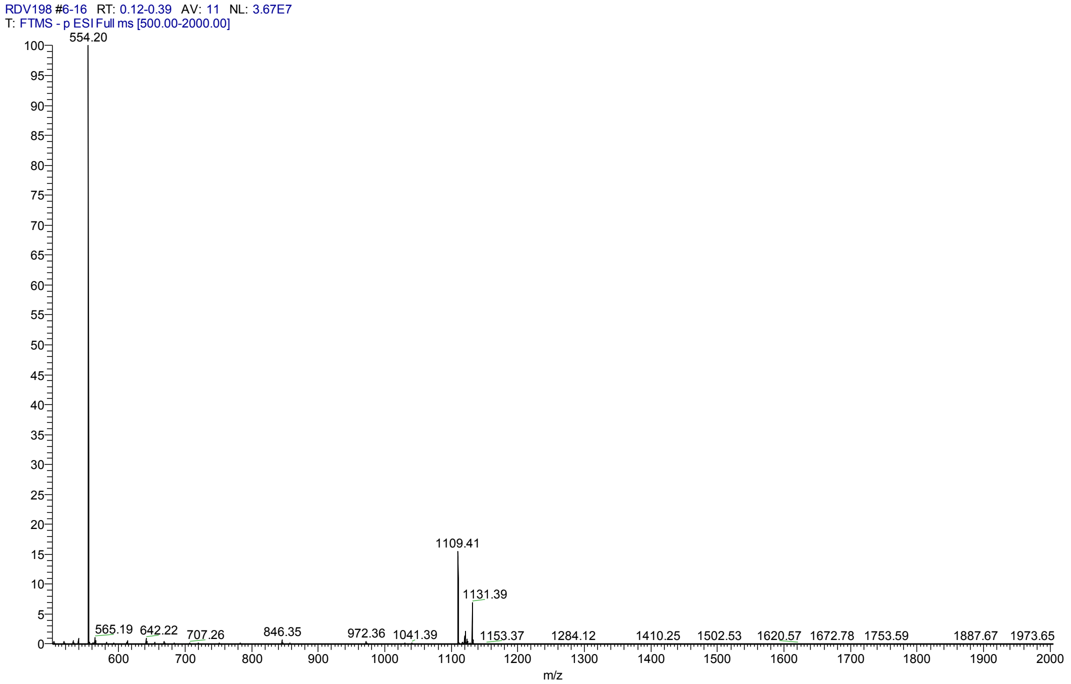

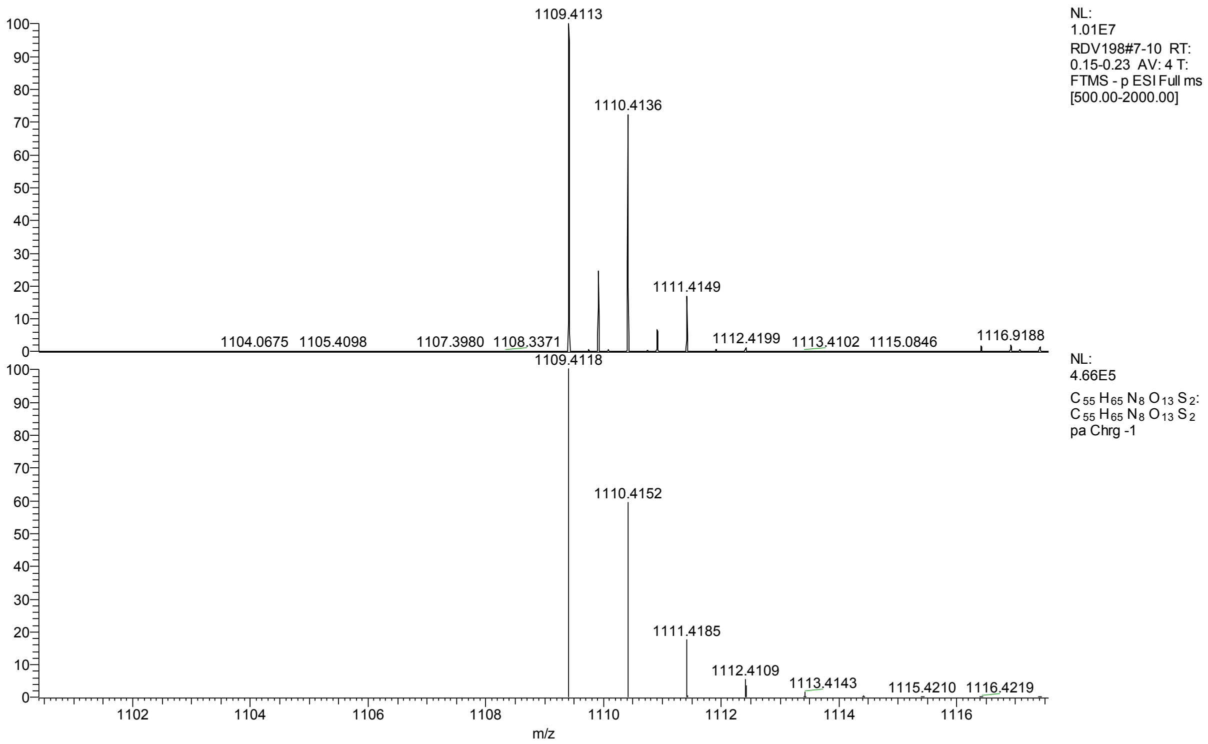
1109.4113
1110.4136
1111.4149
1104.0675
1105.4098
1107.3980
1108.3371
1112.4199
1113.4102
1115.0846
1116.9188
NL:
1.01E7
RDV198#7-10 RT: 0.15-0.23 AV: 4 T: FTMS - p ESI Full ms [500.00-2000.00]
1109.4118
1110.4152
1111.4185
1112.4109
1113.4143
1115.4210
1116.4219
NL:
4.66E5
C 55 H 65 N 8 O 13 S 2: C 55 H 65 N 8 O 13 S 2 pa Chrg -1
m/z

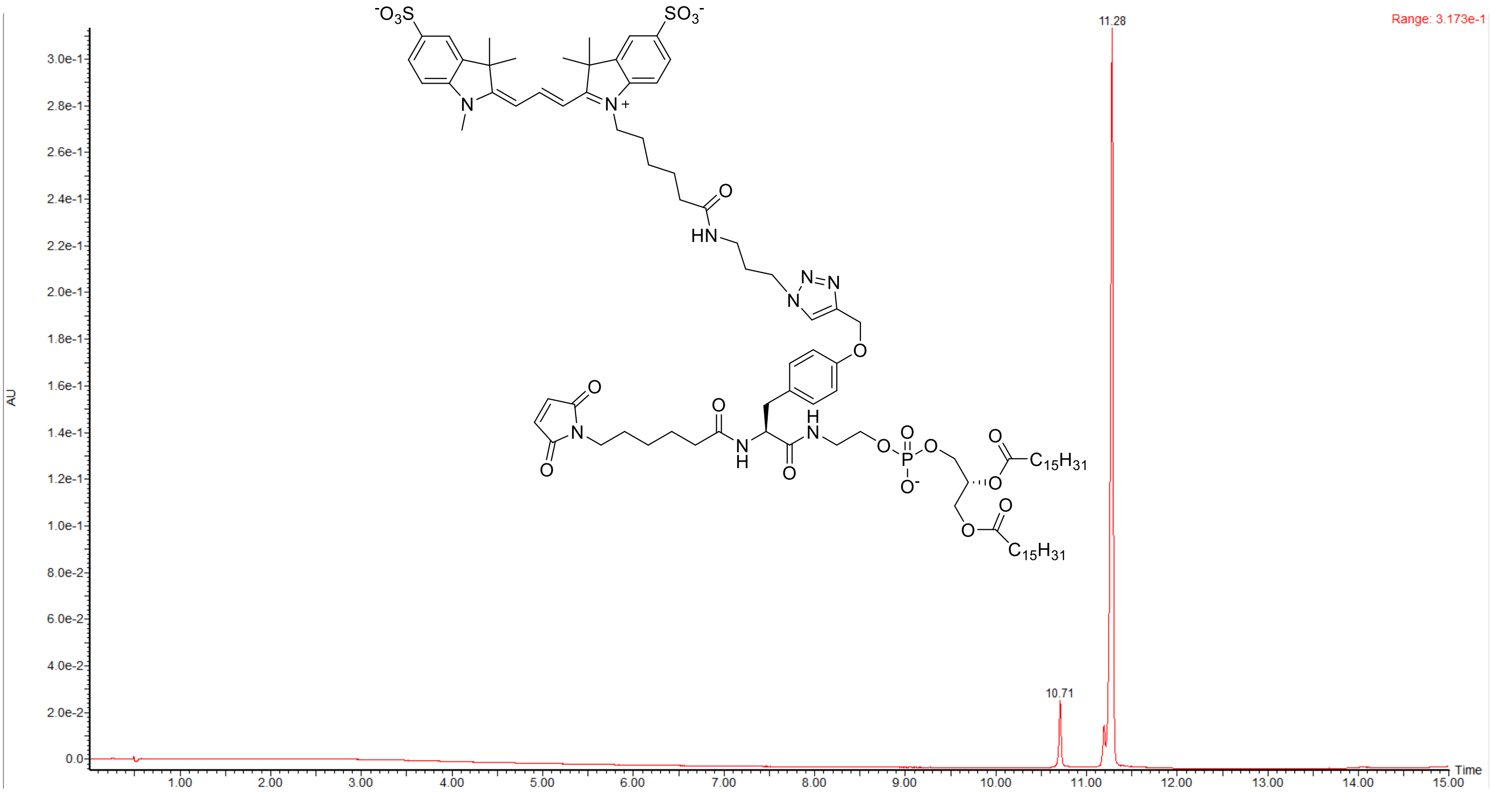
Range: 3.173e-1
11.28
10.71
AU
Time

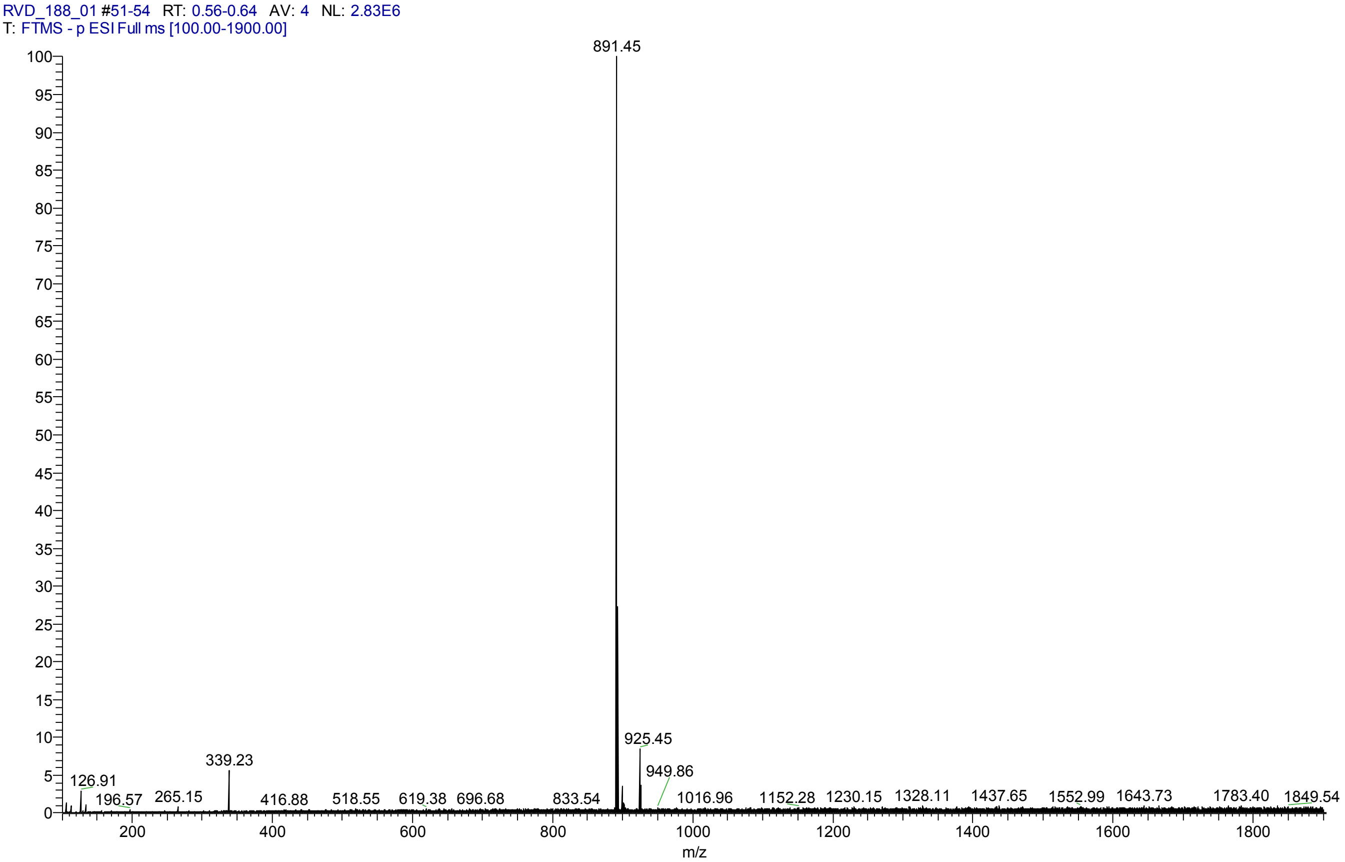
RVD_188_01 #51-54 RT: 0.56-0.64 AV: 4 NL: 2.83E6
T: FTMS - p ESI Full ms [100.00-1900.00]
891.45
925.45
949.86
339.23
126.91
196.57
265.15
416.88
518.55
619.38
696.68
833.54
1016.96
1152.28
1230.15
1328.11
1437.65
1552.99
1643.73
1783.40
1849.54
m/z

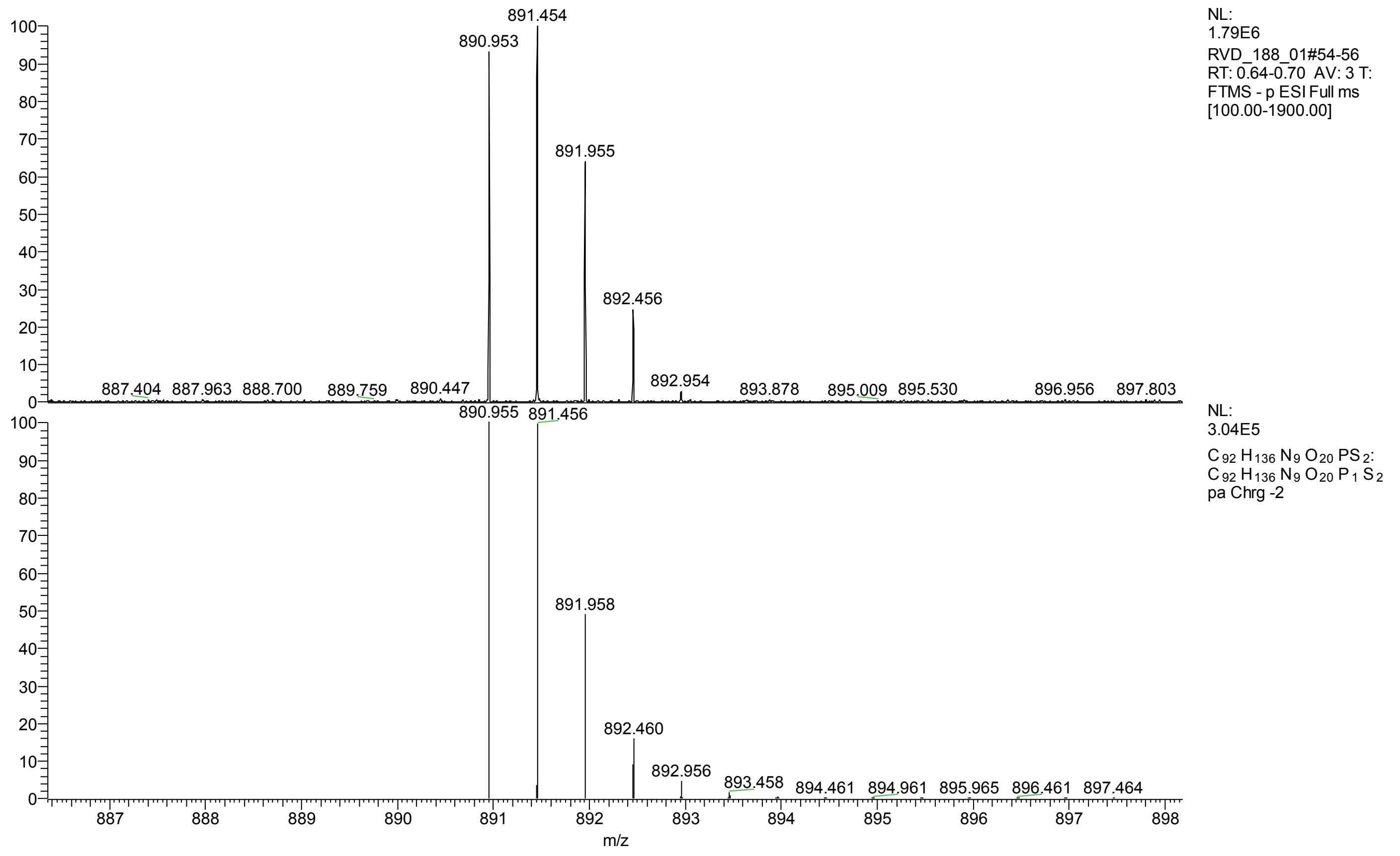
NL:
1.79E6
RVD_188_01#54-56
RT: 0.64-0.70 AV: 3 T:
FTMS - p ESI Full ms
[100.00-1900.00]
891.454
890.953
891.955
892.456
892.954
887.404
887.963
888.700
889.759
890.447
893.878
895.009
895.530
896.956
897.803
NL:
3.04E5
C 92 H 136 N 9 O 20 PS 2:
C 92 H 136 N 9 O 20 P 1 S 2
pa Chrg -2
890.955
891.456
891.958
892.460
892.956
893.458
894.461
894.961
895.965
896.461
897.464
m/z

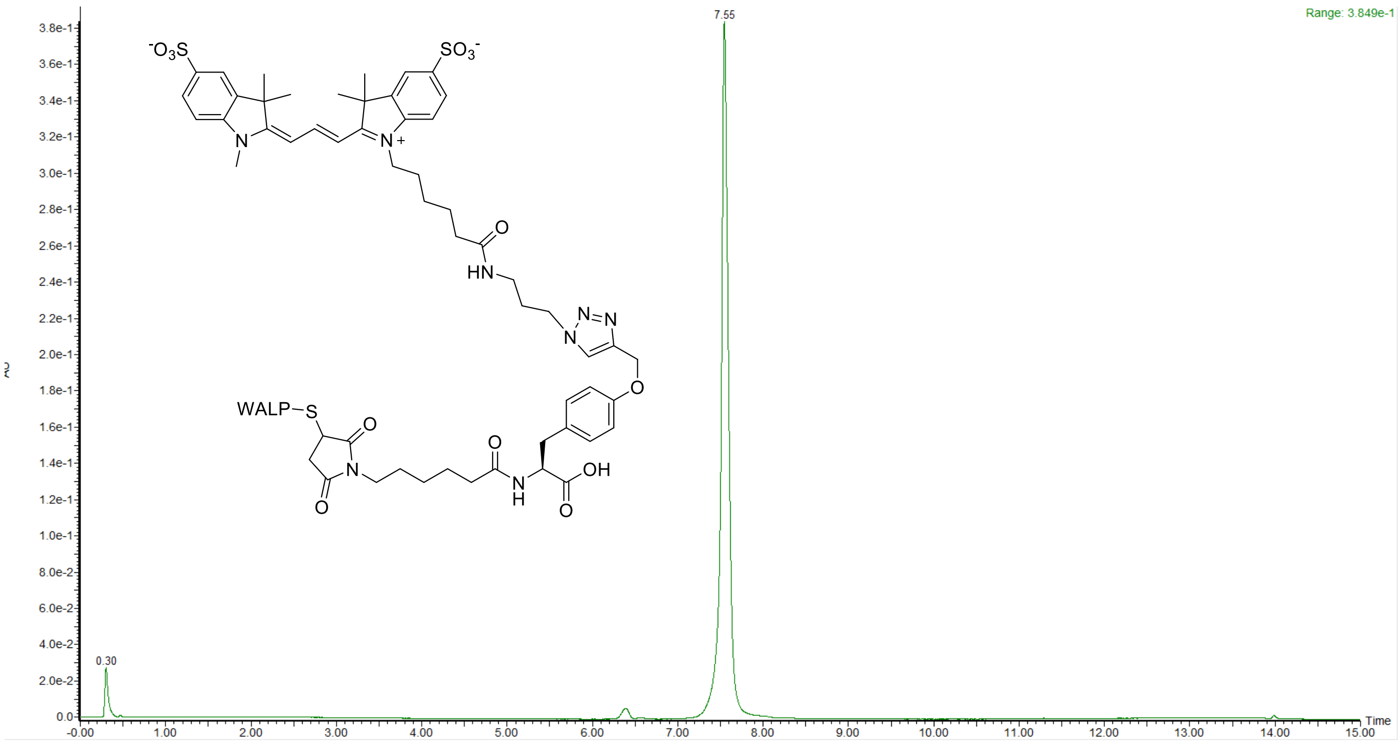
Range: 3.849e-1
7.55
0.30
Time
WALP–S

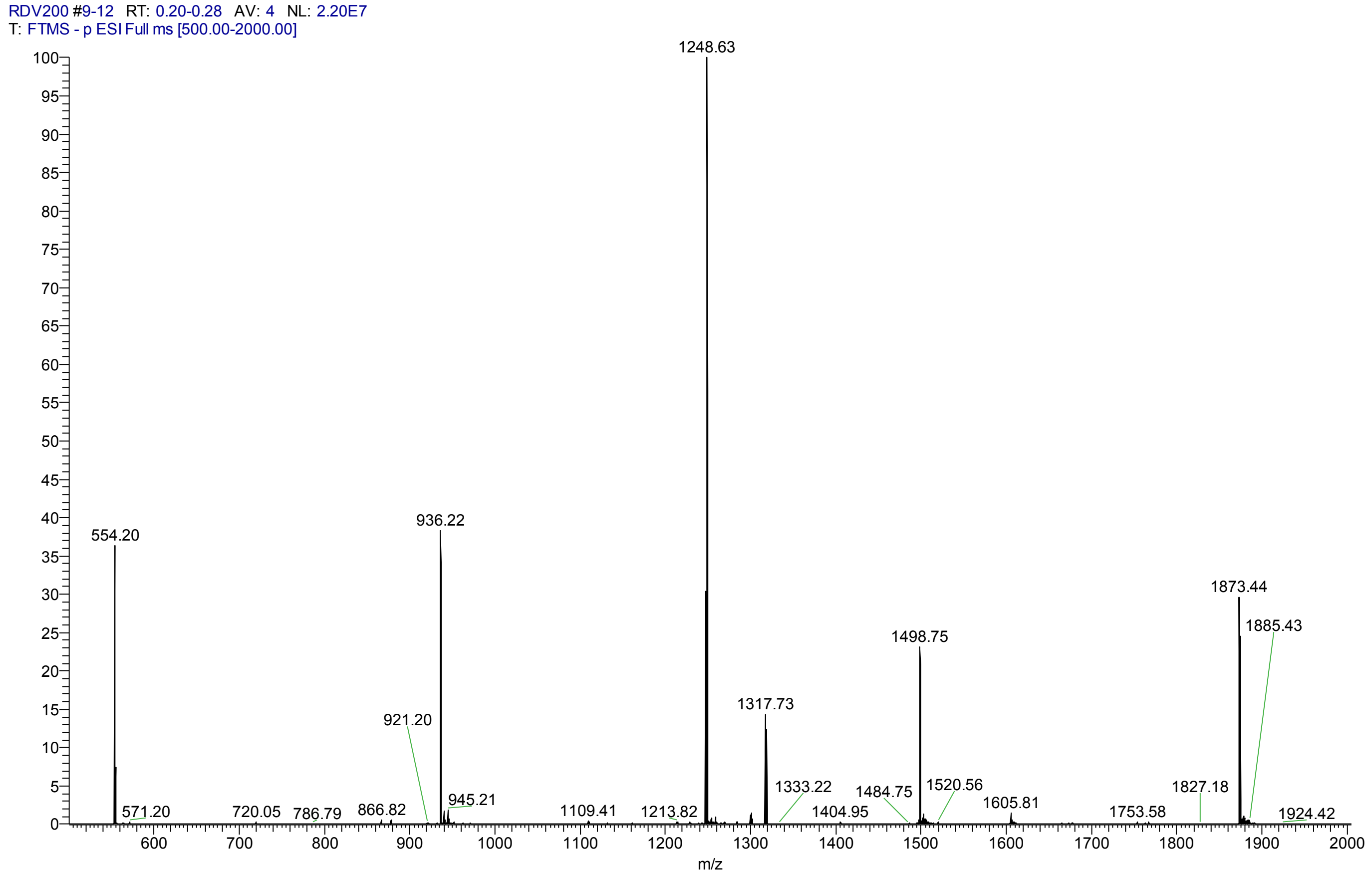
RDV200 #9-12 RT: 0.20-0.28 AV: 4 NL: 2.20E7
T: FTMS - p ESI Full ms [500.00-2000.00]
1248.63
554.20
936.22
921.20
945.21
1317.73
1498.75
1873.44
1885.43
1827.18
1333.22
1484.75
1520.56
1605.81
1404.95
1753.58
1924.42
571.20
720.05
786.79
866.82
1109.41
1213.82
m/z

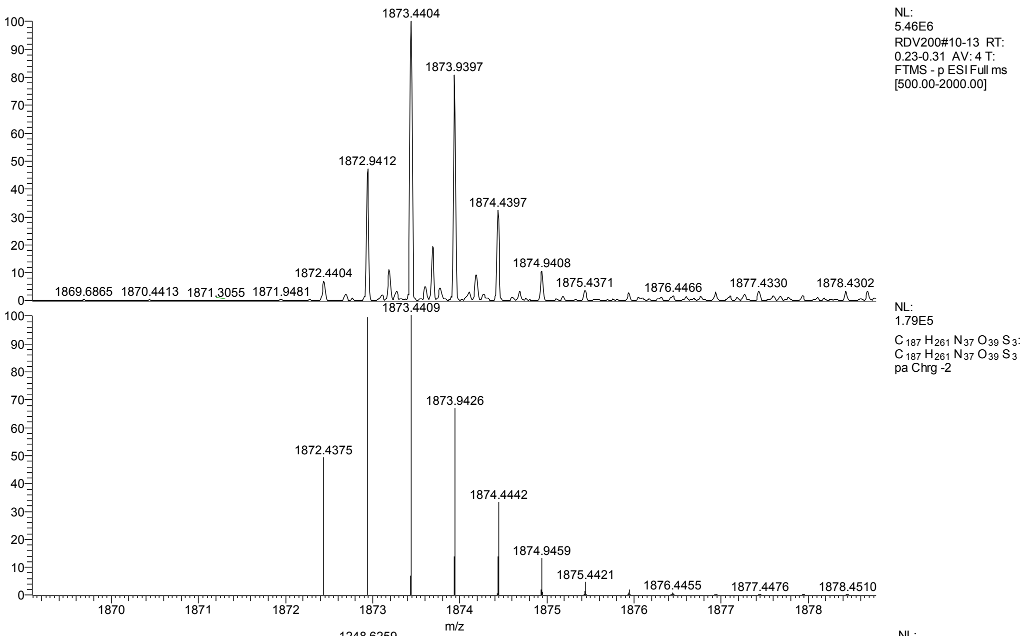
NL:
5.46E6
RDV200#10-13 RT:
0.23-0.31 AV: 4 T:
FTMS - p ESI Full ms
[500.00-2000.00]
NL:
1.79E5
C 187 H 261 N 37 O 39 S 3
C 187 H 261 N 37 O 39 S 3
pa Chrg -2
1873.4404
1873.9397
1872.9412
1874.4397
1874.9408
1872.4404
1875.4371
1876.4466
1877.4330
1878.4302
1869.6865
1870.4413
1871.3055
1871.9481
1873.4409
1873.9426
1872.4375
1874.4442
1874.9459
1875.4421
1876.4455
1877.4476
1878.4510
m/z

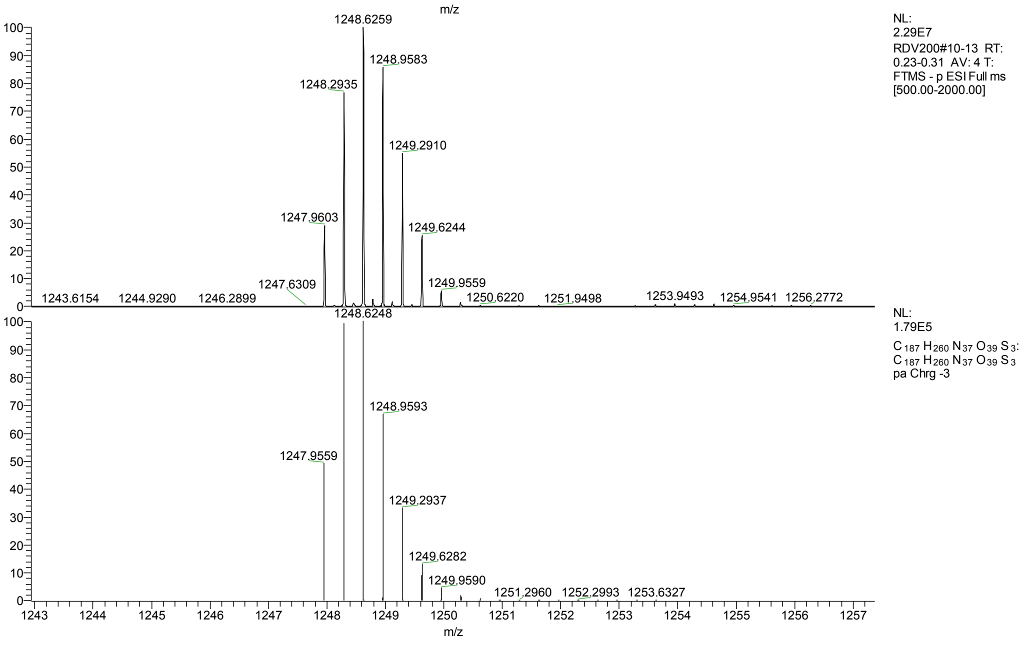
NL:
2.29E7
RDV200#10-13 RT:
0.23-0.31 AV: 4 T:
FTMS - p ESI Full ms
[500.00-2000.00]
NL:
1.79E5
C 187 H 260 N 37 O 39 S 3
C 187 H 260 N 37 O 39 S 3
pa Chrg -3
1248.6259
1248.9583
1248.2935
1249.2910
1247.9603
1249.6244
1249.9559
1247.6309
1243.6154
1244.9290
1246.2899
1250.6220
1251.9498
1253.9493
1254.9541
1256.2772
1248.6248
1248.9593
1247.9559
1249.2937
1249.6282
1249.9590
1251.2960
1252.2993
1253.6327
m/z

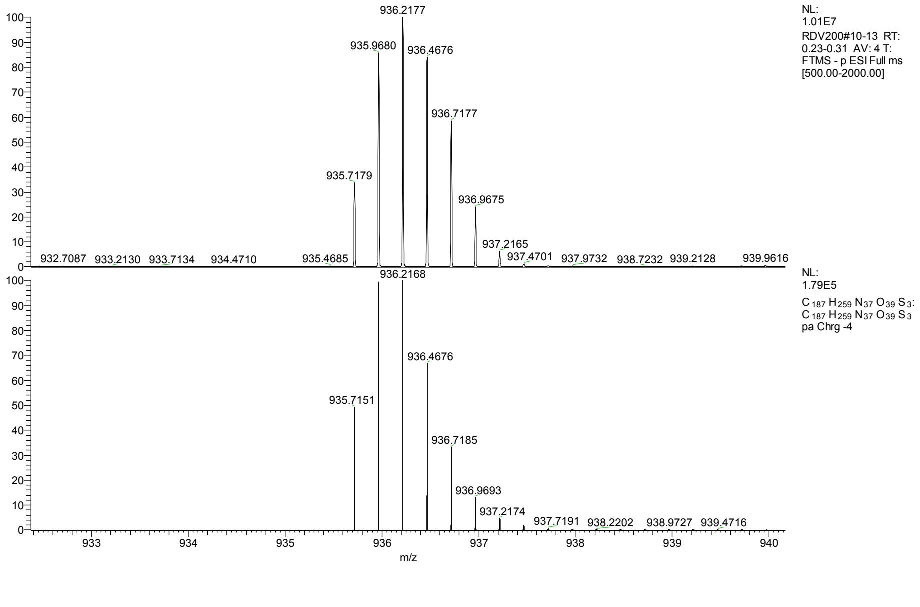
NL:
1.01E7
RDV200#10-13 RT:
0.23-0.31 AV: 4 T:
FTMS - p ESI Full ms
[500.00-2000.00]
NL:
1.79E5
C 187 H 259 N 37 O 39 S 3
C 187 H 259 N 37 O 39 S 3
pa Chrg -4
936.2177
935.9680
936.4676
936.7177
935.7179
936.9675
937.2165
937.4701
932.7087
933.2130
933.7134
934.4710
935.4685
937.9732
938.7232
939.2128
939.9616
936.2168
936.4676
935.7151
936.7185
936.9693
937.2174
937.7191
938.2202
938.9727
939.4716
m/z

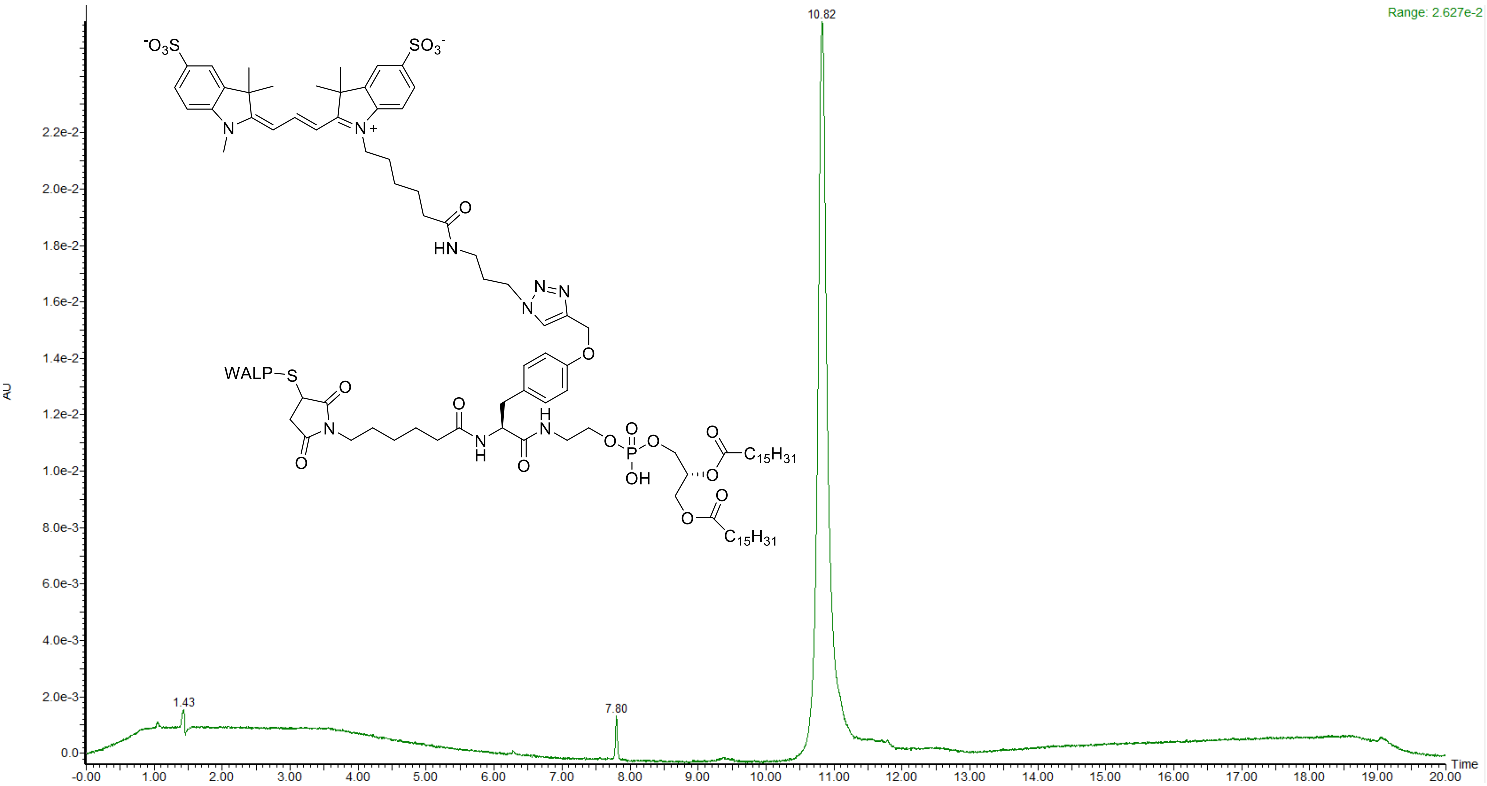
10.82
Range: 2.627e-2
AU
WALP
1.43
7.80
Time

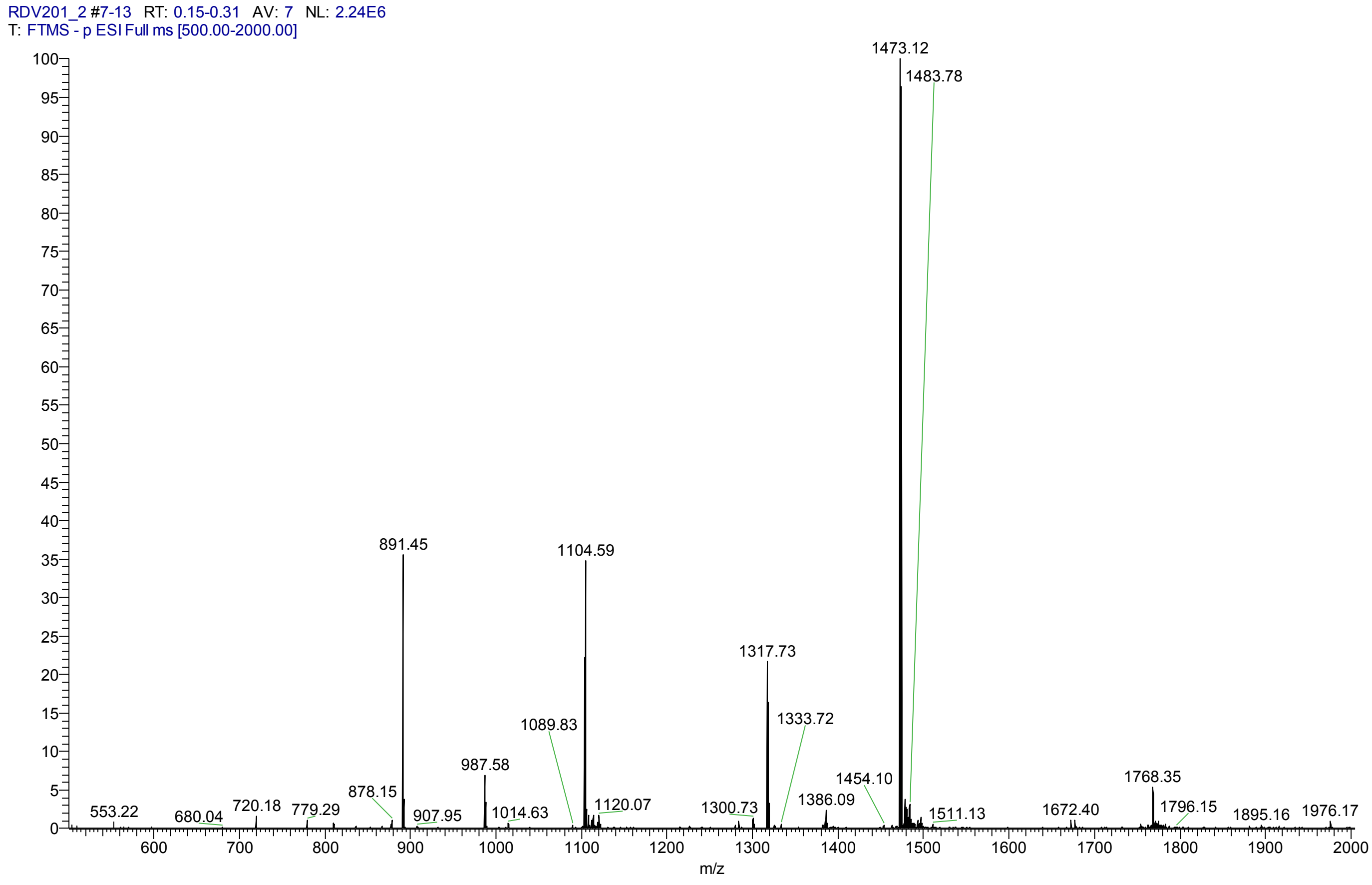
RDV201_2 #7-13 RT: 0.15-0.31 AV: 7 NL: 2.24E6
T: FTMS - p ESI Full ms [500.00-2000.00]
1473.12
1483.78
891.45
1104.59
1317.73
1333.72
1089.83
987.58
1454.10
1768.35
878.15
720.18
779.29
1120.07
1300.73
1386.09
553.22
680.04
907.95
1014.63
1511.13
1672.40
1796.15
1895.16
1976.17
m/z

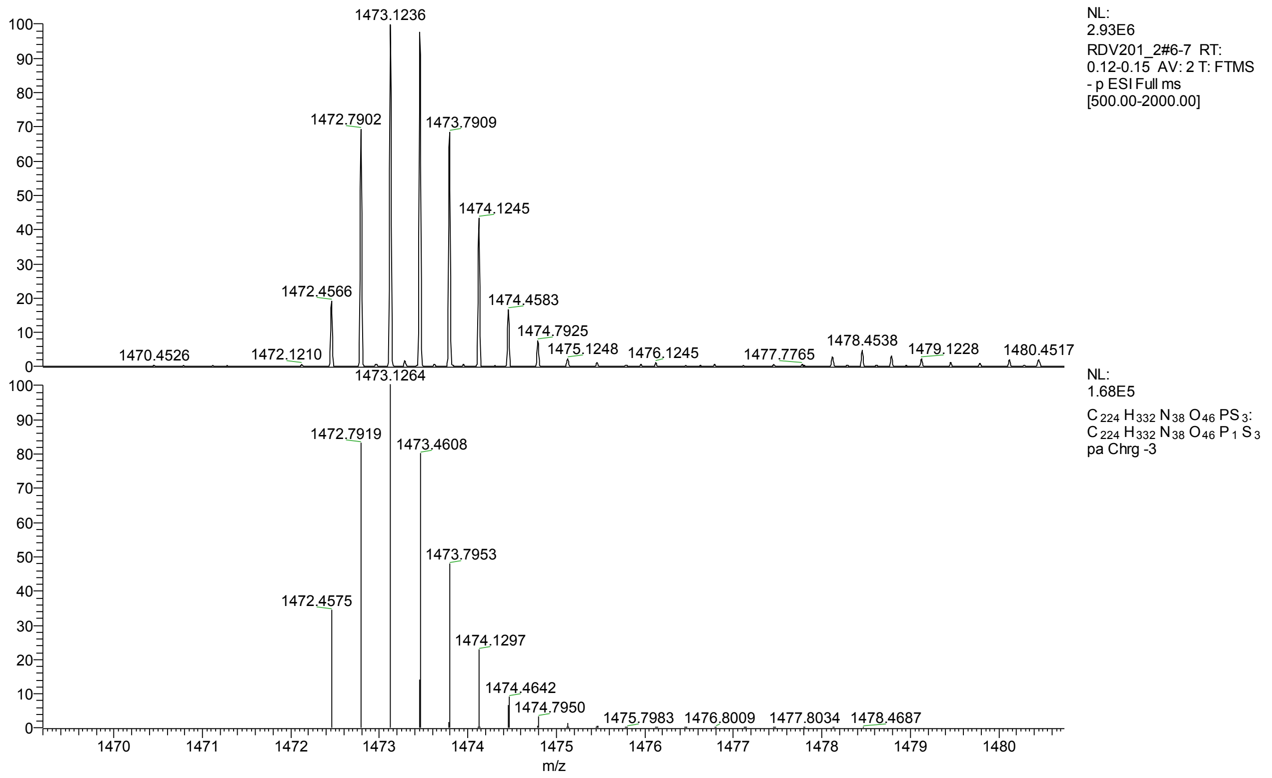
NL:
2.93E6
RDV201_2#6-7 RT:
0.12-0.15 AV: 2 T: FTMS
- p ESI Full ms
[500.00-2000.00]
NL:
1.68E5
C 224 H 332 N 38 O 46 PS 3:
C 224 H 332 N 38 O 46 P 1 S 3
pa Chrg -3
m/z

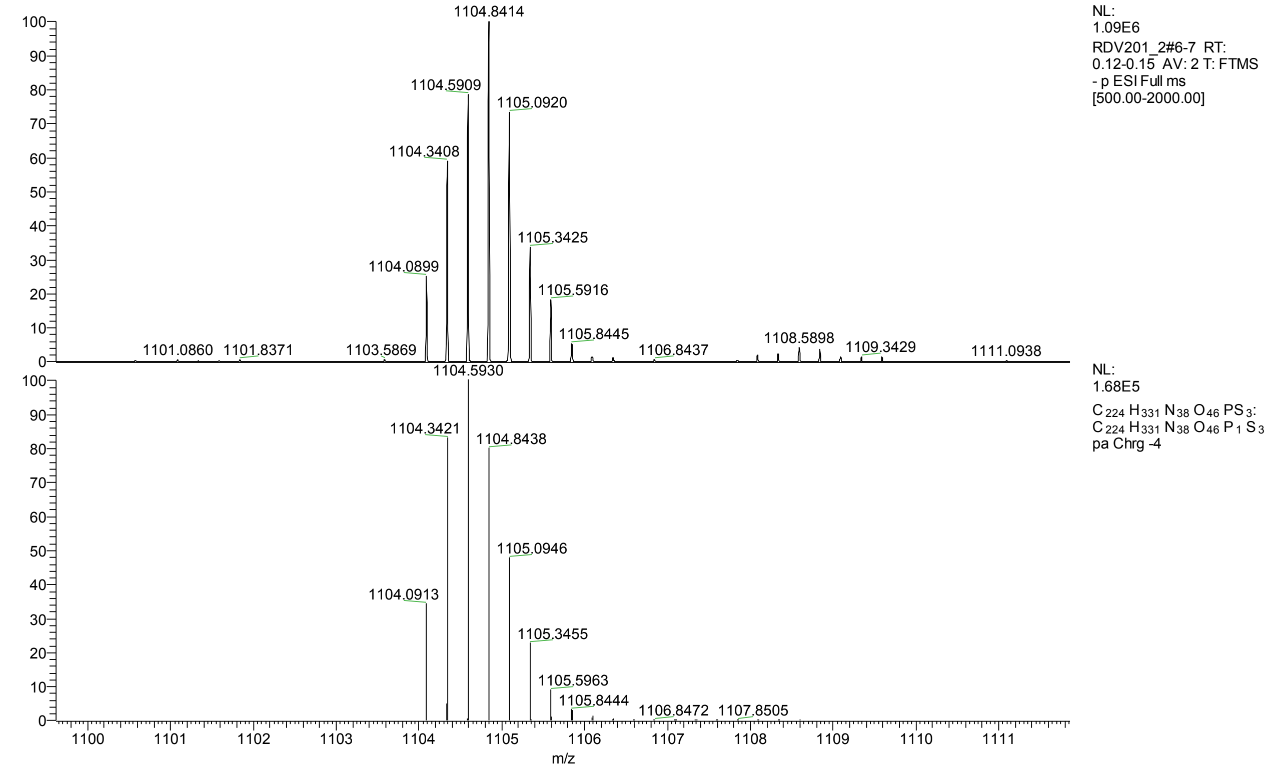
NL:
1.09E6
RDV201_2#6-7 RT:
0.12-0.15 AV: 2 T: FTMS
- p ESI Full ms
[500.00-2000.00]
NL:
1.68E5
C 224 H 331 N 38 O 46 PS 3:
C 224 H 331 N 38 O 46 P 1 S 3
pa Chrg -4
m/z
